# PGLYRP2 drives hepatocyte-intrinsic innate immunity by trapping and clearing hepatitis B virus

**DOI:** 10.1172/JCI188083

**Published:** 2025-02-13

**Authors:** Ying Li, Huihui Ma, Yongjian Zhang, Tinghui He, Binyang Li, Haoran Ren, Jia Feng, Jie Sheng, Kai Li, Yu Qian, Yunfeng Wang, Haoran Zhao, Jie He, Huicheng Li, Hongjin Wu, Yuanfei Yao, Ming Shi

**Affiliations:** 1 School of Life Science and Technology, Harbin Institute of Technology, Harbin, China.; 2 International Research Center for Regenerative Medicine, Boao International Hospital, Qionghai, China.; 3 Biomedical Postgraduate Workstation of Heilongjiang Province, Harbin, China.; 4 Department of Surgery Oncology, Sixth Affiliated Hospital of Harbin Medical University, Harbin, China.; 5 Department of Surgery Oncology, Harbin Medical University Cancer Hospital, Harbin, China.; 6 School of Medicine and Health, Harbin Institute of Technology, Harbin, China.; 7 Harbin Pharmaceutical Group Bioengineering Company, Harbin, China.; 8 School of Life and Health Sciences, Hainan University, Haikou, China.; 9 Department of Medical Oncology, Harbin Medical University Cancer Hospital, Harbin, China.

**Keywords:** Hepatology, Virology, Cellular immune response, Hepatitis

## Abstract

Spontaneous clearance of hepatitis B virus (HBV) is frequent in adults (95%) but rare in infants (5%), emphasizing the critical role of age-related hepatic immunocompetence. However, the underlying mechanisms of hepatocyte-specific immunosurveillance and age-dependent HBV clearance remain unclear. Here, we identified PGLYRP2 as a hepatocyte-specific pattern recognition receptor with age-dependent expression, and demonstrated that phase separation of PGLYRP2 was a critical driver of spontaneous HBV clearance in hepatocytes. Mechanistically, PGLYRP2 recognized and potentially eliminated covalently closed circular DNA via phase separation, coordinated by its intrinsically disordered region and HBV DNA-binding domain (PGLYRP2^IDR/209–377^) in the nucleus. Additionally, PGLYRP2 suppressed HBV capsid assembly by directly interacting with the viral capsid, mediated by its PGRP domain. This interaction promoted the nucleocytoplasmic translocation of PGLYRP2 and subsequent secretion of the PGLYRP2/HBV capsid complex, thereby bolstering the hepatic antiviral response. Pathogenic variants or deletions in PGLYRP2 impaired its ability to inhibit HBV replication, highlighting its essential role in hepatocyte-intrinsic immunity. These findings suggest that targeting the PGLYRP2-mediated host-virus interaction may offer a potential therapeutic strategy for the development of anti-HBV treatments, representing a promising avenue for achieving a functional cure for HBV infection.

## Abstract 2

Human PGLYRP2 is identified as a crucial innate molecule mediating HBV clearance in hepatocytes by targeting both viral cccDNA and capsid assembly, and by modulating host antiviral immune responses.

## Graphical abstract

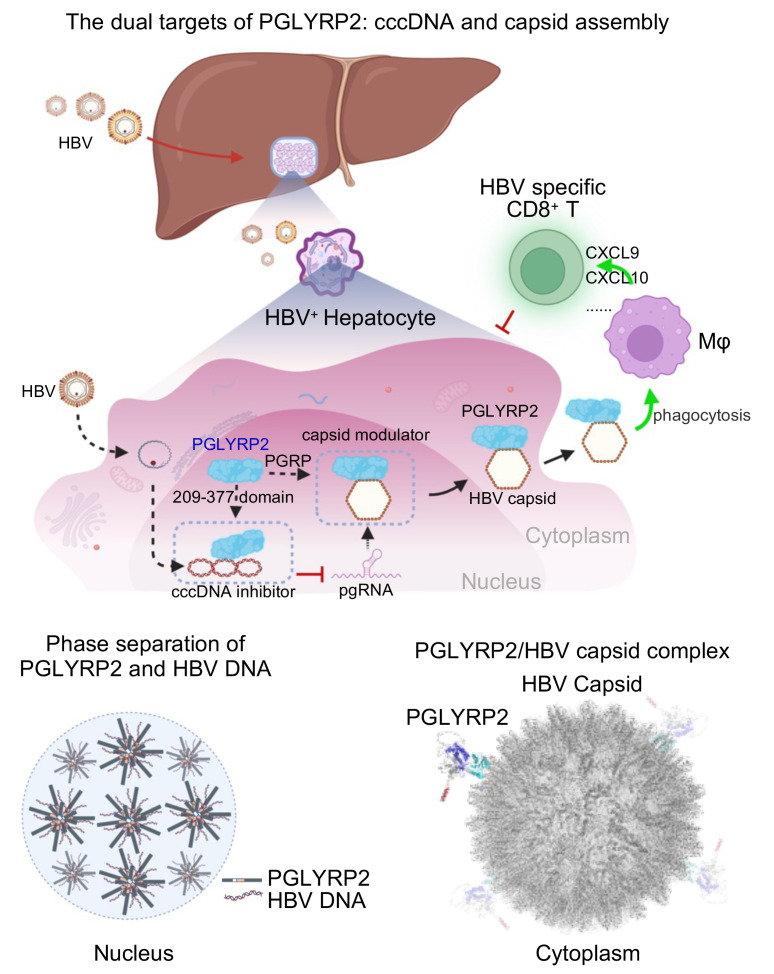

## Introduction

Chronic hepatitis B virus (HBV) infection poses a substantial challenge to global health, precipitating severe clinical outcomes including liver failure, cirrhosis, and hepatocellular carcinoma (1–3). Despite the availability of prophylactic vaccines aimed at reducing HBV incidence, no therapeutic strategies currently eradicate the virus in the approximately 296 million individuals affected worldwide (4). The susceptibility to this chronic condition is predominantly mediated by the viral entry receptor in hepatocytes, the human sodium taurocholate cotransporting polypeptide (hNTCP). Variability in the host’s viral clearance mechanisms, which is substantially influenced by age, plays a crucial role in combating HBV infection (5–8). Addressing these age-related variations is essential for developing targeted therapies that enhance hepatic immune competence, crucial in managing HBV effectively.

Historically, HBV has been regarded as a “stealth” virus, believed to evade the innate immune response at initial infection — a viewpoint supported by earlier studies (9, 10). However, recent evidence challenges this perspective, demonstrating that human primary hepatocytes and myeloid cells can detect HBV particles via innate sensors during the initial stages of infection (11–15). Moreover, hepatocytes are essential in orchestrating systemic innate immunity, secreting innate immune molecules whose activity and effectiveness are markedly influenced by age (16–20).

Peptidoglycan recognition protein 2 (PGLYRP2), synthesized predominantly by hepatocytes and circulating in the bloodstream of healthy individuals, has emerged as a pivotal innate sensor and immunomodulator (21, 22). It has been previously implicated in the modulation of the tumor microenvironment and proposed as a biomarker for robust antitumor immune responses (23). This study further investigates the intricate interactions between PGLYRP2 and HBV within hepatocytes and their impact on the hepatic microenvironment. Our findings reveal the critical antiviral role of PGLYRP2 as an age-dependent HBV scavenger that targets both viral covalently closed circular DNA (cccDNA) and nucleocapsids, thus bolstering intrinsic defense mechanisms in hepatocytes. These insights underscore the potential of PGLYRP2 as a robust anti-HBV agent, capable of inhibiting both viral cccDNA and capsid assembly, making targeting of PGLYRP2 a promising avenue for achieving effective control and a functional cure of HBV infection.

## Results

### Identification of PGLYRP2 as an age-dependent HBV DNA-binding protein.

**Figure 1:**
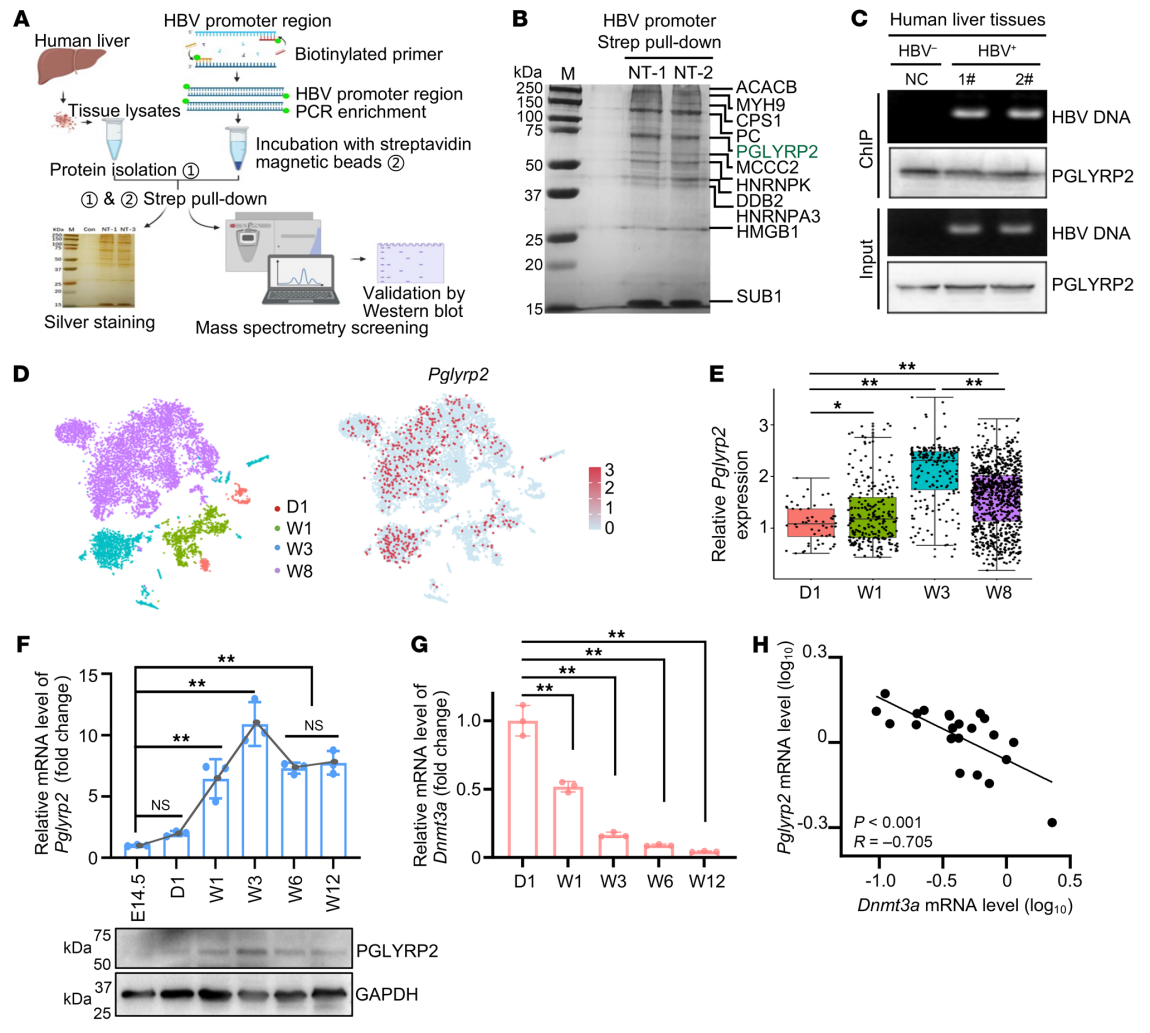
Age-dependent expression of PGLYRP2 and its interactions with HBV promoter DNA. (**A**) Schematic overview of screening for HBV promoter–binding proteins. Diagram illustrating the experimental design for identifying proteins that bind to HBV promoter regions. (**B**) Identification of HBV promoter–binding host proteins. Proteins bound to HBV promoter DNA were isolated using a biotin-streptavidin affinity pull-down assay and visualized on a 12% SDS-PAGE gel with silver staining to confirm purity and presence. (**C**) ChIP assays were conducted to examine the interaction between PGLYRP2 and HBV promoter DNA. Anti-PGLYRP2 antibodies were used to pull down the relevant DNA-protein complexes from HBV^+^ and HBV^–^ tissue lysates (Input). (**D**) Single-cell transcriptomic analysis by t-distributed stochastic neighbor embedding (t-SNE). Left: t-SNE plots illustrating the distribution of hepatocytes sampled at 4 distinct time points, displayed in 4 different colors. Right: A separate t-SNE plot highlights expression levels of PGLYRP2 across these cells. D, day; W, week. (**E**) Bar plots showing expression levels of PGLYRP2 in hepatocytes, as derived from the t-SNE analysis. The *y* axis represents log-normalized expression levels, emphasizing differences across time points. (**F**) Age-related expression of *Pglyrp2* in mouse liver. Real-time PCR and Western blot analyses were used to measure PGLYRP2 levels across various age groups in mouse liver, illustrating an age-dependent expression pattern. (**G**) *Dnmt3a* expression analysis in mouse liver by age group. Real-time PCR was used to assess the expression of *Dnmt3a* across different age groups, revealing a decline in expression with age. Dots indicate biological replicates (*n* = 3 independent experiments). (**H**) Correlation between DNMT3A and PGLYRP2 expression. The relationship between *Dnmt3a* and *Pglyrp2* mRNA levels was quantitatively analyzed in mouse liver tissues, highlighting a significant negative correlation. Data are represented as mean ± SD. Kruskal-Wallis with Dunn’s post hoc multiple-comparison test (**E**) and 1-way ANOVA with post hoc Bonferroni’s test (**F** and **G**) were used for statistical analysis. Pearson’s correlation coefficient was used in **H**. *P < 0.05; **P < 0.001.

To gain deeper insights into the regulation of HBV replication, we aimed to identify host proteins in human hepatocytes that interact with HBV promoter DNA, using a biotin-streptavidin affinity pull-down assay. Whole-cell lysates from normal liver tissues of human paracancerous tissues (NT) were incubated with streptavidin magnetic beads coupled to 5′ biotinylated HBV promoter DNA probes containing enhancer I (Enh I), basic core promoter (BCP)/Enh II, and negative regulatory element (NRE). The proteins that bound to these HBV promoter regions were isolated from the lysates and subsequently analyzed by mass spectrometry (Figure 1A).

Among the HBV promoter DNA interactome, characterized predominantly by DNA- or RNA-binding proteins, including the previously reported HBV cccDNA host factors YBX1 (24, 25), HMGB1 (26, 27), and DDB2 (28, 29), the HCV regulators HNRNPK (30, 31) and HNRNPA3 (31), and the potential HIV-associated protein SUB1 (32), our attention was drawn to PGLYRP2, an innate immune molecule that is highly and constitutively expressed in the liver and predominantly localized within the nuclei of hepatocytes (23, 33) (Figure 1B and Supplemental Table 1; supplemental material available online with this article; https://doi.org/10.1172/JCI188083DS1). Using chromatin immunoprecipitation (ChIP) assays, we robustly confirmed the interaction between PGLYRP2 and HBV promoter DNA, demonstrating its presence in both human liver tissues and established cell lines. This pivotal interaction, depicted in Figure 1C and further elucidated in Supplemental Figure 1A, highlights the potential regulatory influence of PGLYRP2 on HBV transcriptional dynamics, reinforcing its role in the molecular orchestration of HBV infection control.

In light of the age-related hepatic immune competence crucial for HBV clearance, we meticulously examined the expression dynamics of *PGLYRP2*. Using a comprehensive single-cell RNA sequencing (scRNA-Seq) dataset of mice (34), we discerned distinct hepatocyte clusters primarily differentiated by their collection intervals. Initial observations revealed a notably sparse expression of *Pglyrp2* on day 1, with a subsequent significant augmentation from week 1 through week 8, as illustrated in Figure 1, D and E. This temporal expression pattern was rigorously validated through quantitative reverse transcription PCR and Western blot analyses on isolated mouse hepatocytes, aligning with the observed age-dependent expression trends (Figure 1F). Furthermore, the age-related upregulation of hepatic *PGLYRP2* expression was substantiated in human liver tissues via analysis of RNA-Seq datasets, providing a robust framework for understanding its role in modulating immune competence against HBV across different age groups (35, 36) (Supplemental Figure 1, B and C).

Considering that DNA methyltransferase 3A (DNMT3A) functioned in mammalian development and was responsible for the downregulation of *PGLYRP2* (23, 33), we evaluated the mRNA levels of mouse *Dnmt3a* across 5 age groups and analyzed its correlation with *Pglyrp2* levels. The transcript levels of *Dnmt3a* showed a significant decline with age (*P* < 0.001; Figure 1G), and a negative expression correlation was observed between *Dnmt3a* and *Pglyrp2* in mouse liver tissues (*P* < 0.001; *R* = –0.7934, 95% CI: –0.9066 to –0.5736; Figure 1H). We also characterized the methylation status of the *Pglyrp2* promoter region using bisulfate sequencing, which revealed hypermethylation at embryonic day 14.5 (E14.5) but reduced methylation from week 1 (W1) to week 12 (W12) (Supplemental Figure 1D). Significantly, levels of *Pglyrp2* were markedly elevated after treatment of E14.5 hepatocytes with the DNA demethylating agent 5-Aza-CdR (Supplemental Figure 1E).

Hepatic differentiation markers are crucial for understanding the relationship between *PGLYRP2* expression and the developmental status of liver tissues. Our analysis, using data from The Cancer Genome Atlas (TCGA) and quantitative reverse transcription PCR, explored the association between *PGLYRP2* expression and levels of hepatic differentiation markers, including *AFP*, *ALB*, *APOF*, *CPS1*, *DPP4*, *HNF4A*, and *TTR*, in human normal tumor-adjacent liver tissues. The results indicate that *PGLYRP2* expression correlates with these hepatic differentiation markers (Supplemental Table 2 and Supplemental Figure 1, F and G), suggesting an age-related expression profile for *PGLYRP2*.

### PGLYRP2 acts as a cccDNA inhibitor to limit HBV replication.

**Figure 2:**
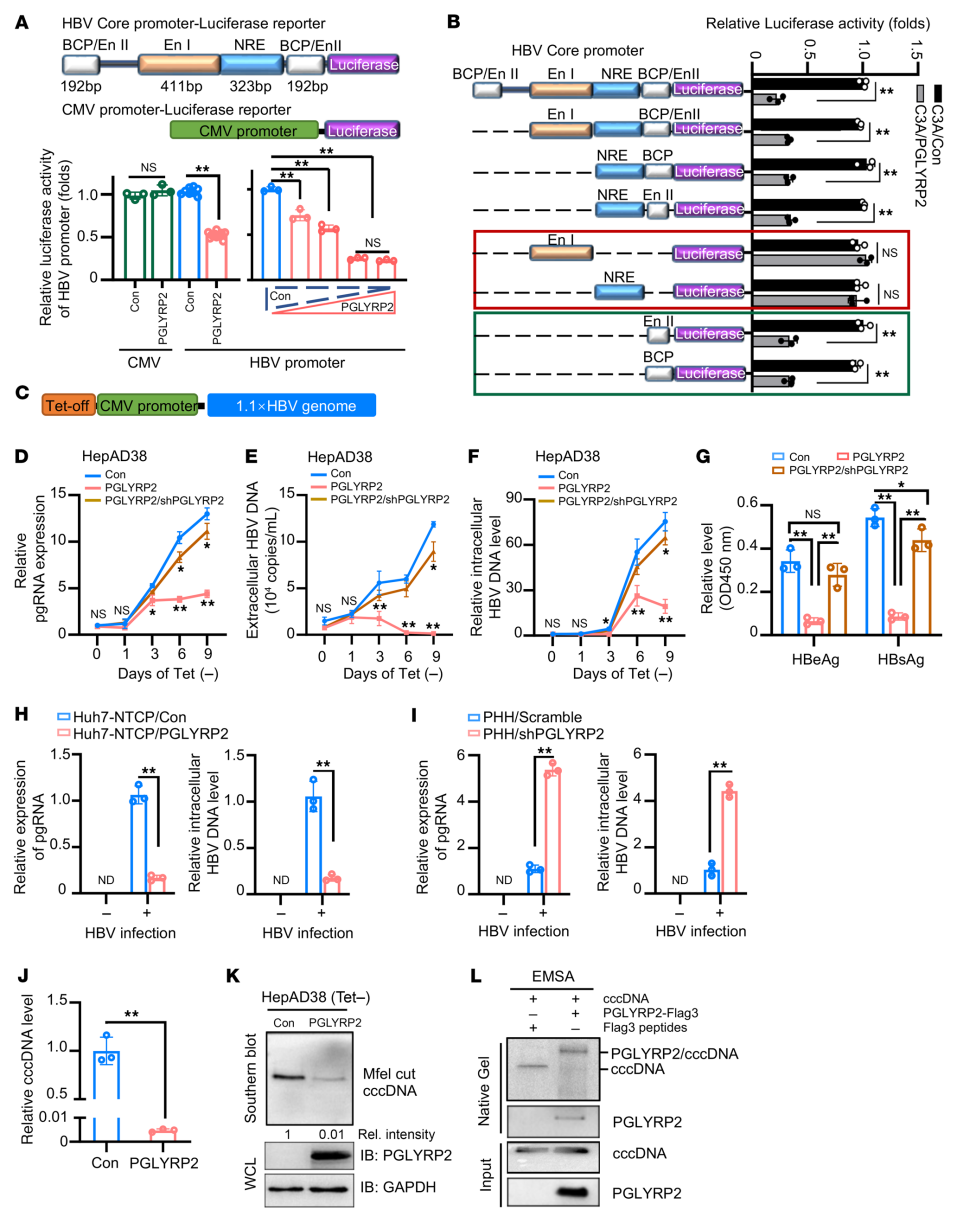
Hepatocyte-derived PGLYRP2 inhibited HBV core promoter activity and cccDNA formation. (**A**) Schematic representation depicts the HBV promoter–luciferase reporter construct containing BCP/Enh II, Enh I, and NRE regions, alongside a CMV promoter–driven luciferase reporter for control purposes (top). The cotransfection setup in C3A cells (bottom) involves varying concentrations of PGLYRP2 expression plasmid (0–400 ng) or a control vector, alongside a Renilla luciferase reporter to assess transfection efficiency. Dots indicate biological replicates (*n* = 3–12; independent experiments). (**B**) Truncated HBV promoter constructs assessed for luciferase activities with PGLYRP2 cotransfection. Dots indicate biological replicates (right, *n* = 3). (**C**) Schematic representation of HBV genome (1.1 copies) under a Tet-off CMV promoter in HepAD38 cells. (**D**–**G**) Monitoring HBV replication in modified HepAD38 cell lines. HepAD38 lines (control, PGLYRP2 expressing,or PGLYRP2 knockdown) either control vector, PGLYRP2, or shRNA against PGLYRP2 were cultured in Tet-free conditions for 9 days. Intracellular pgRNA (**D**), extracellular/ intracellular HBV DNA (**E** and **F**), and supernatant HBeAg/HBsAg (**G**, ELISA) were analyzed. (**H** and **I**) HBV infection assay in modified Huh7-NTCP and PHH cells (control or PGLYRP2-suppressed). Levels of intracellular pgRNA and HBV DNA after infection were analyzed (day 9; *n* = 3). (**J**) cccDNA quantification in HepAD38 cells treated with ExoI/ExoIII/T5 nuclease (day 9 in Tet-free medium). PCR (**J**; n = 3) and Southern blot (**K**, normalized to control). Western blot shows PGLYRP2/GAPDH levels. (**L**) Evaluation of cccDNA binding activity by EMSA. Purified PGLYRP2 or control peptides were incubated with Mfei-cut cccDNA, resolved via native agarose gel, and analyzed by Southern and Western blotting. Data are represented as mean ± SD. Two-tailed Student’s *t* test (**A**, left), 1-way ANOVA with post hoc Bonferroni’s test (**A**, right, and **G**), 2-way ANOVA with post hoc Bonferroni’s test (**D**–**F**), and 2-tailed Student’s *t* test (**B** and **H**–**J**) were used for statistical analysis. *P < 0.05; **P < 0.001.

To elucidate the mechanisms underlying age-related hepatic immunocompetence in combating HBV infection, we explored the regulatory role of PGLYRP2 in viral RNA transcription by binding to HBV promoter regions using a luciferase reporter assay. Notably, PGLYRP2 significantly reduced HBV promoter–driven luciferase activity in a dose-dependent manner, while CMV promoter–driven luciferase expression remained unaffected (Figure 2A and Supplemental Figure 2A). To determine the specific regulatory regions of PGLYRP2 within the HBV promoter, we constructed truncated HBV promoter–luciferase reporter plasmids. Our findings indicated that the BCP/Enh II regions were pivotal for PGLYRP2-mediated suppression of HBV promoter activity, whereas the Enh I and NRE regions were not involved in this suppression (Figure 2B and Supplemental Figure 2B).

In the HepAD38 cell system, where pregenome RNA (pgRNA) expression is controlled by a tetracycline-responsive (Tet-responsive) element (Figure 2C), we cultivated stable cell lines HepAD38/Con, HepAD38/PGLYRP2, and HepAD38/PGLYRP2/shPGLYRP2 in Tet-free medium for 9 days. We observed that intracellular pgRNA levels in HepAD38/PGLYRP2 cells were significantly suppressed from day 3 to day 9, in contrast to the increased pgRNA levels observed in HepAD38/Con and HepAD38/PGLYRP2/shPGLYRP2 cells (Figure 2D). This pattern suggests dual regulation of pgRNA transcription by both CMV and physiological HBV promoters in HepAD38 cells, achieving a balance between CMV-driven HBV expression and PGLYRP2-mediated viral suppression (Figure 2C). This hypothesis was confirmed in experiments using cells transfected with either CMV or physiological HBV promoters, where PGLYRP2 significantly inhibited the transcriptional activity of the physiological HBV promoters (Supplemental Figure 2, C and D).

Further, the suppression of both intracellular and extracellular HBV DNA, as well as the production of supernatant HBeAg and HBsAg by PGLYRP2, was observed in HepAD38/PGLYRP2 cells in the absence of Tet (Figure 2, E–G, and Supplemental Figure 2E). Remarkably, extracellular HBV DNA was almost completely inhibited by PGLYRP2 within 6–9 days following Tet withdrawal (Figure 2E). These findings were corroborated using the stable cell line and an ex vivo cultivated primary human hepatocyte (PHH)–HBV infection system (Figure 2, H and I, and Supplemental Figure 2, F and G).

To elucidate the mechanism of nuclear PGLYRP2-mediated HBV suppression, Hirt DNA was extracted from HepAD38/PGLYRP2 cultured in Tet-free medium, and the cccDNA levels were evaluated using Southern blot and real-time PCR methods. Interestingly, the data showed that cccDNA formation was almost abrogated in Tet-free medium–cultured HepAD38/PGLYRP2 (Figure 2, J and K, and Supplemental Figure 2H), demonstrating that PGLYRP2 reduces the cccDNA level in HBV-infected hepatocytes. EMSA assay further determined that PGLYRP2 could bind with cccDNA (Figure 2L). Collectively, these results suggest that hepatocyte-specific PGLYRP2 has an effect on immunosurveillance of HBV infection in the nucleus and restricts HBV replication and cccDNA formation by binding to HBV BCP/Enh II promoter regions.

### PGLYRP2^IDR^-mediated phase separation serves as a platform for the trapping of HBV DNA by PGLYRP2^209–377^.

**Figure 3:**
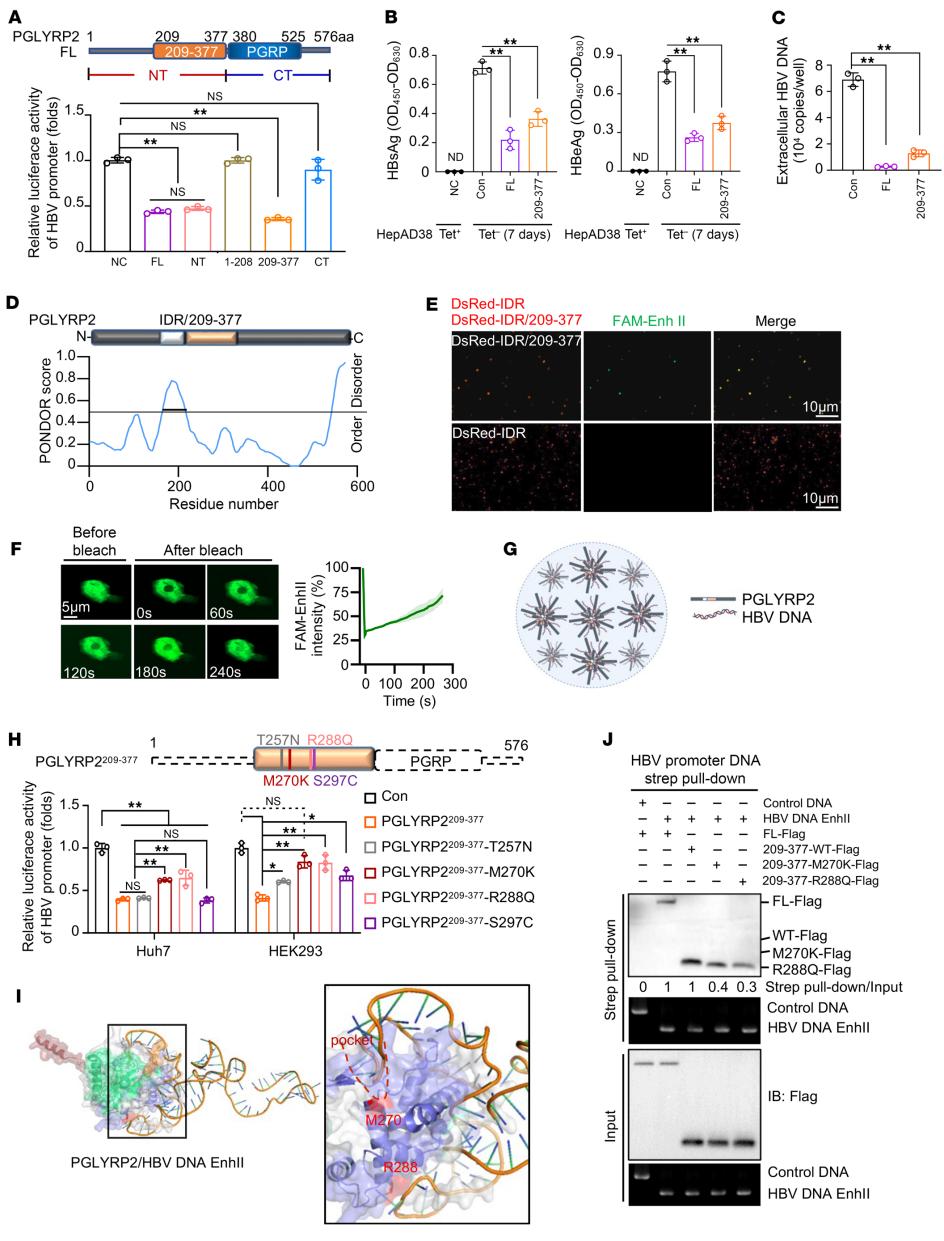
Role of PGLYRP2^IDR^-mediated phase separation in trapping HBV DNA and impact of pathogenic variants in human PGLYRP2^209–377^ on viral inhibition. (**A**–**C**) Schematic representation and functional assays of PGLYRP2 variants. (**A**) Top: Schematic representation of full-length (FL) and truncated forms of PGLYRP2. Bottom: Cotransfection assays in C3A cells used HBV promoter–luciferase reporter constructs with either FL or truncated PGLYRP2 plasmids, alongside HBV or HBc expression constructs. (**B**) ELISA and PCR analysis in stable HepAD38 cell lines for supernatant levels of HBsAg and HBeAg. (**C**) Real-time PCR determined the levels of intracellular HBV DNA. (**D**) Prediction of intrinsically disordered regions in PGLYRP2 using the PONDR tool. (**E**) Expression and phase separation analysis of DsRed-tagged PGLYRP2^IDR/209–377^ and DsRed-tagged PGLYRP2^IDR^. DsRed-PGLYRP2^IDR/209–377^ facilitated the formation of membraneless condensates that colocalized with HBV DNA FAM–Enh II. Scale bars: 10 μm. (**F**) FRAP analysis of HBV DNA FAM–Enh II within PGLYRP2^IDR/209–377^-induced condensates. Right: Quantification of fluorescence intensity. Scale bars: 5 μm. (**G**) Model of PGLYRP2-mediated phase separation as a platform for trapping HBV DNA. (**H**) Relative luciferase activity analysis of PGLYRP2 variants with missense SNPs. (**I**) Structural and protein-DNA docking analyses. Conformations of PGLYRP2 and HBV DNA Enh II were predicted using AlphaFold3 and UNAFold, respectively. Protein-DNA docking with HDOCK elucidated specific interactions between the bent structure of Enh II and the PGLYRP2^209–377^ pocket. (**J**) HBV promoter DNA pull-down assay. Cell lysates from HEK293 cells transfected with FL PGLYRP2, WT, or SNP-containing PGLYRP2^209–377^ constructs were incubated with 5′ biotinylated HBV promoter DNA probes or control DNA and streptavidin-agarose beads. Data are represented as mean ± SD. One-way ANOVA with post hoc Bonferroni’s test was used for statistical analysis (**A**–**C** and **H**). *P < 0.05; **P < 0.001.

To delineate the functional domain of PGLYRP2 crucial for inhibiting HBV, we engineered several truncations of the protein. The HBV promoter–luciferase reporter assay indicated that both the full-length PGLYRP2 and truncations containing the PGLYRP2^209–377^ domain significantly diminished HBV promoter activity, suggesting a critical role for this segment (Figure 3A). Moreover, HepAD38 cells expressing either the full-length protein or the PGLYRP2^209–377^ domain exhibited markedly reduced levels of supernatant HBsAg, HBeAg, and extracellular HBV DNA, reinforcing the domain’s importance in viral suppression (Figure 3, B and C).

The intrinsically disordered region (IDR) of PGLYRP2, predicted using PONDR (Predictor of Natural Disordered Regions), spans amino acids 164–208 and is adjacent to the PGLYRP2^209–377^ domain (Figure 3D). Purified DsRed-tagged PGLYRP2^IDR/209–377^ triggers the formation of membraneless condensates that colocalize with HBV DNA FAM–Enh II. Meanwhile, condensates induced by DsRed-tagged PGLYRP2^IDR^ do not include HBV DNA FAM–Enh II, suggesting that phase separation induced by PGLYRP2^IDR^ might underpin the viral suppression function of PGLYRP2^209–377^ (Figure 3E). Furthermore, we analyzed the dynamics of HBV DNA FAM–Enh II within PGLYRP2^IDR/209–377^-induced condensates using fluorescence recovery after photobleaching (FRAP). The fluorescence of FAM–Enh II puncta recovered slowly after photobleaching (Figure 3F and Supplemental Video 1), indicating that PGLYRP2, by inducing phase separation involving HBV DNA, likely sequesters the viral DNA from cellular machinery, thereby potentially obstructing its role in viral replication (Figure 3G).

### Pathogenic variants in human PGLYRP2^209–377^ disrupt the HBV inhibition.

We further investigated pathogenic single-nucleotide variants (SNVs) within the human PGLYRP2^209–377^ domain, as identified in the 1000 Genomes Project and TCGA Project. Notably, several SNVs with a genotype frequency greater than 0.005 were found, including single-nucleotide polymorphism (SNP) T257N (rs28404490, 1000 Genomes minor allele frequency [1000G MAF] = 0.039); SNP M270K (rs892145, 1000G MAF = 0.365), which has been associated with Parkinson’s disease; SNP R288Q (rs149627417, 1000G MAF = 0.0067); and SNP S297C (TCGA Liver Hepatocellular Carcinoma, allele frequency = 0.1) (37–39). We investigated the impact of these missense SNVs on PGLYRP2-mediated viral suppression. Among these SNVs, M270K and R288Q variants resulted in the highest HBV promoter–luciferase activity, suggesting that these mutations compromise viral suppression efficacy (Figure 3H). Structural analyses using AlphaFold3 (https://deepmind.google/technologies/alphafold/alphafold-server/) and UNAFold (https://www.unafold.org/) predicted the conformations of PGLYRP2 and HBV DNA Enh II, respectively. Subsequent protein-DNA docking conducted with HDOCK (http://hdock.phys.hust.edu.cn/) highlighted a specific interaction: the distinctively bent structure of Enh II fits into a pocket at residue M270 of PGLYRP2, while the positively charged R288 interacts with Enh II (Figure 3I and Supplemental Figure 3). Biotin-streptavidin affinity pull-down assays targeting HBV Enh II further demonstrated a reduced binding capacity of the PGLYRP2^209–377^ domain to HBV DNA when mutated at M270K or R288Q, substantiating the domain’s pivotal role in mediating effective HBV inhibition (Figure 3J).

### Nucleocytoplasmic translocation of PGLYRP2 triggered by interaction with HBV capsid.

**Figure 4:**
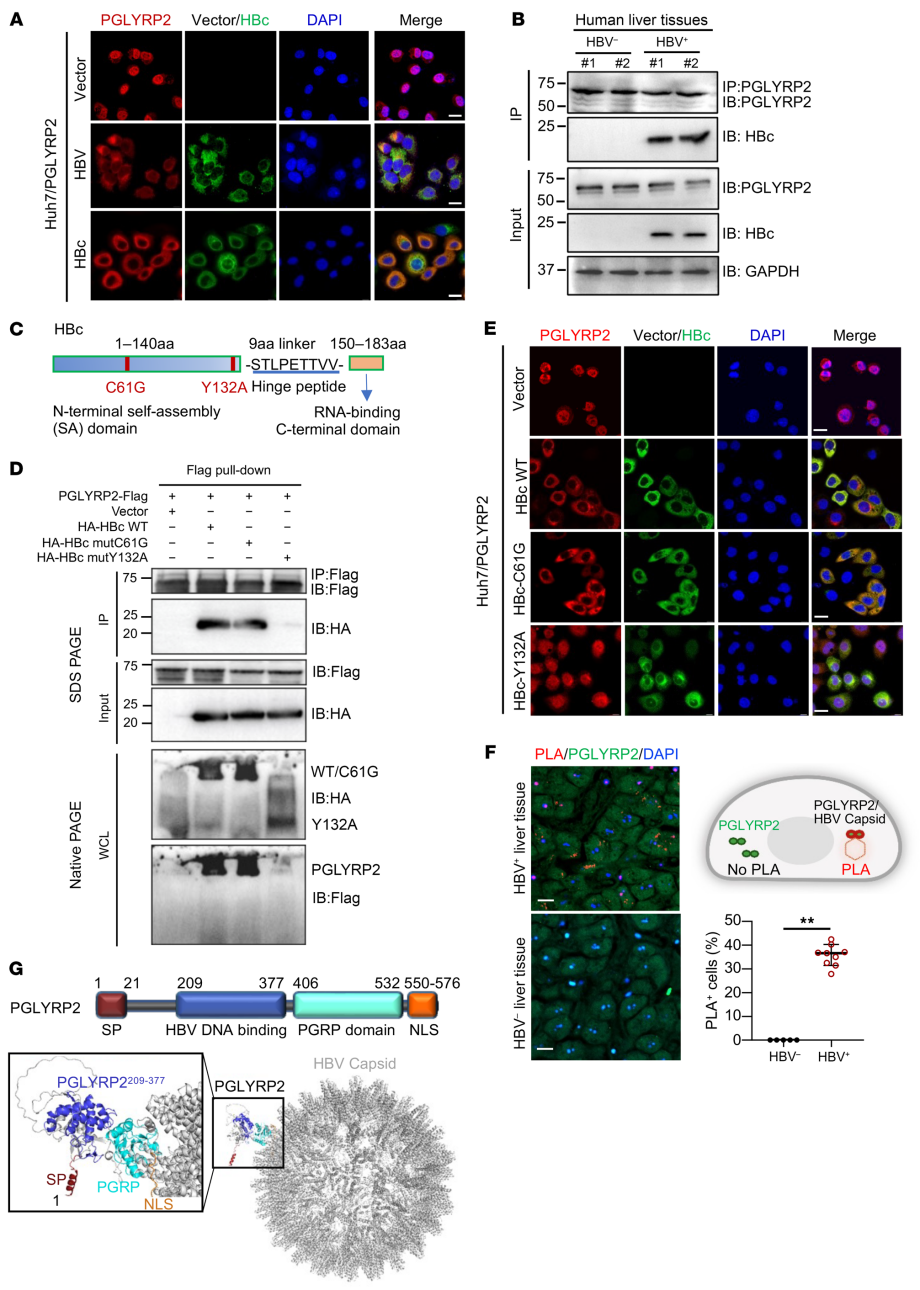
HBV capsid interacts with PGLYRP2 and induces nucleocytoplasmic translocation in HBV-infected cells. (**A**) Localization of PGLYRP2 (red) and HBc (green) in HBV or HBc expression construct–transfected Huh7/PGLYRP2 cells was analyzed by immunofluorescence staining. Scale bars: 20 μm. (**B**) HBV^–^ or HBV^+^ human liver tissue lysates were prepared for co-IP using anti-PGLYRP2 and blotted using the indicated antibodies. (**C**) Schematic representation of WT and mutants of HBc (C61G and Y132A). (**D**) PGLYRP2 expression construct was cotransfected with Con vector, HBc WT, HBc-C61G, or HBc-Y132A expression construct into HEK293 cells. After 48 hours, cell lysates were harvested for co-IP using anti-FLAG antibody and blotted using the indicated antibodies. The high-order complex in the whole-cell lysates (WCLs) was separated by native PAGE and subjected to immunoblot. (**E**) Nucleocytoplasmic translocation of PGLYRP2 (red) in WT HBc, HBc-C61G, or HBc-Y132A mutant (green) expression construct–transfected Huh7/PGLYRP2 cells was analyzed by immunofluorescence staining. Scale bars: 20 μm. (**F**) Left: Representative confocal images showing PLA^+^ and PLA^–^ signal in HBV^+^ liver tissue or HBV^–^ liver tissue, respectively. Top right: A PLA signal corresponds to PGLYRP2/HBV capsid proximity, whereas the absence of HBV capsid resulted in the lack of a PLA fluorescent signal. Bottom right: Quantification of percentage of PLA^+^ cells from the total number of detected cells. Dots indicate biological replicates (*n* = 5 for HBV^–^ tissues and *n* = 9 for HBV^+^ tissues; independent experiments). Scale bars: 20 μm. Data are represented as mean ± SD. Two-tailed Student’s *t* test was used for statistical analysis. **P < 0.001. (**G**) Our proposed model of PGLYRP2^NLS^ masking. The AlphaFold3-predicted structure model of PGLYRP2 and the 3D structure of PGLYRP2/HBV capsid (Protein Data Bank: 6VZP) complex were visualized by PyMOL software (https://pymol.org).

PGLYRP2 predominantly resides in the nucleus in its resting state (23). Upon transfection of Huh7/PGLYRP2 stable cell lines with 1.3 copies of the HBV genome or an HBc expression construct, we observed nucleocytoplasmic translocation of PGLYRP2 in cells expressing HBc, indicating that the viral core protein HBc is a crucial factor in the translocation process (Figure 4A). Analysis of nuclear and cytosolic fractions from whole-cell lysates of Huh7 cells transfected with the 1.3×HBV genome construct, either control or expressing PGLYRP2, further confirmed HBc-induced nucleocytoplasmic translocation of PGLYRP2 (Supplemental Figure 4A).

Investigating factors influencing the nucleocytoplasmic translocation of PGLYRP2 in response to HBV infection, we hypothesized that HBV proteins counteract the host’s intrinsic antiviral responses (40, 41), serving as binding partners for innate sensor PGLYRP2. A FLAG pull-down assay showed that HBc bound to PGLYRP2 with relatively high intensity, whereas HBs bound with lower intensity (Supplemental Figure 4B). Coimmunoprecipitation (co-IP) assays confirmed the interaction between endogenous PGLYRP2 and HBc in HBV-infected human liver tissues (Figure 4B). Pull-down assays with both full-length and truncated PGLYRP2 constructs identified the PGRP domain as the primary binding site for HBc (Supplemental Figure 4, C and D).

HBc exhibits assembly polymorphism, making it challenging for co-IP assays to discern whether PGLYRP2 binds to hexameric HBc (planar trimers of dimers), the high-order HBc oligomer (HBV capsid), or both. To clarify these interactions, we used wild-type (WT) HBc, an HBc hexamer mutant with defective capsid assembly (HBc-Y132A), and an assembly-active HBc mutant (HBc-C61G) (42–44) for our studies (Figure 4C). Despite similar expression levels of HBc-Y132A, HBc-C61G, and WT HBc, native PAGE and Western blot assays revealed that PGLYRP2 coimmunoprecipitated with WT HBc and HBc-C61G, but not with HBc-Y132A (Figure 4D). These results indicate a preference for PGLYRP2 binding to the intact HBV capsid.

To further confirm that the nucleocytoplasmic location was induced by the HBV capsid rather than hexameric HBc, immunofluorescence assays were conducted in Huh7/PGLYRP2 cells transfected with WT HBc, HBc-C61G, or HBc-Y132A mutants. As anticipated, only WT HBc and the assembly-active HBc-C61G mutant induced the translocation, while the assembly-defective HBc-Y132A mutant did not (Figure 4E). A proximity ligation assay (PLA) in HBV^+^ human liver tissue showed a significant proximity between PGLYRP2 and the HBV capsid in the cytosol, confirming the direct interaction within infected cells (Figure 4F).

To elucidate the underlying mechanisms of PGLYRP2 translocation in response to HBV infection, a structural model of the PGLYRP2/HBV capsid complex was generated using AlphaFold3 predictions. The model indicated that the interaction between PGLYRP2^PGRP^ and the capsid may inhibit the activity of the nuclear localization signal (NLS) at the C-terminus of PGLYRP2 as a result of steric hindrance. This interaction enhances the nucleocytoplasmic translocation and subsequent secretion of PGLYRP2, a process facilitated by the signal peptide (SP) at the N-terminus (Figure 4G and Supplemental Figure 4, E and F). A similar phenomenon, wherein the translocation behavior of proteins is influenced by steric hindrance, has been reported in previous studies (45, 46).

### PGLYRP2 translocation in response to HBV infection enhances viral clearance through capsid assembly modulation.

**Figure 5:**
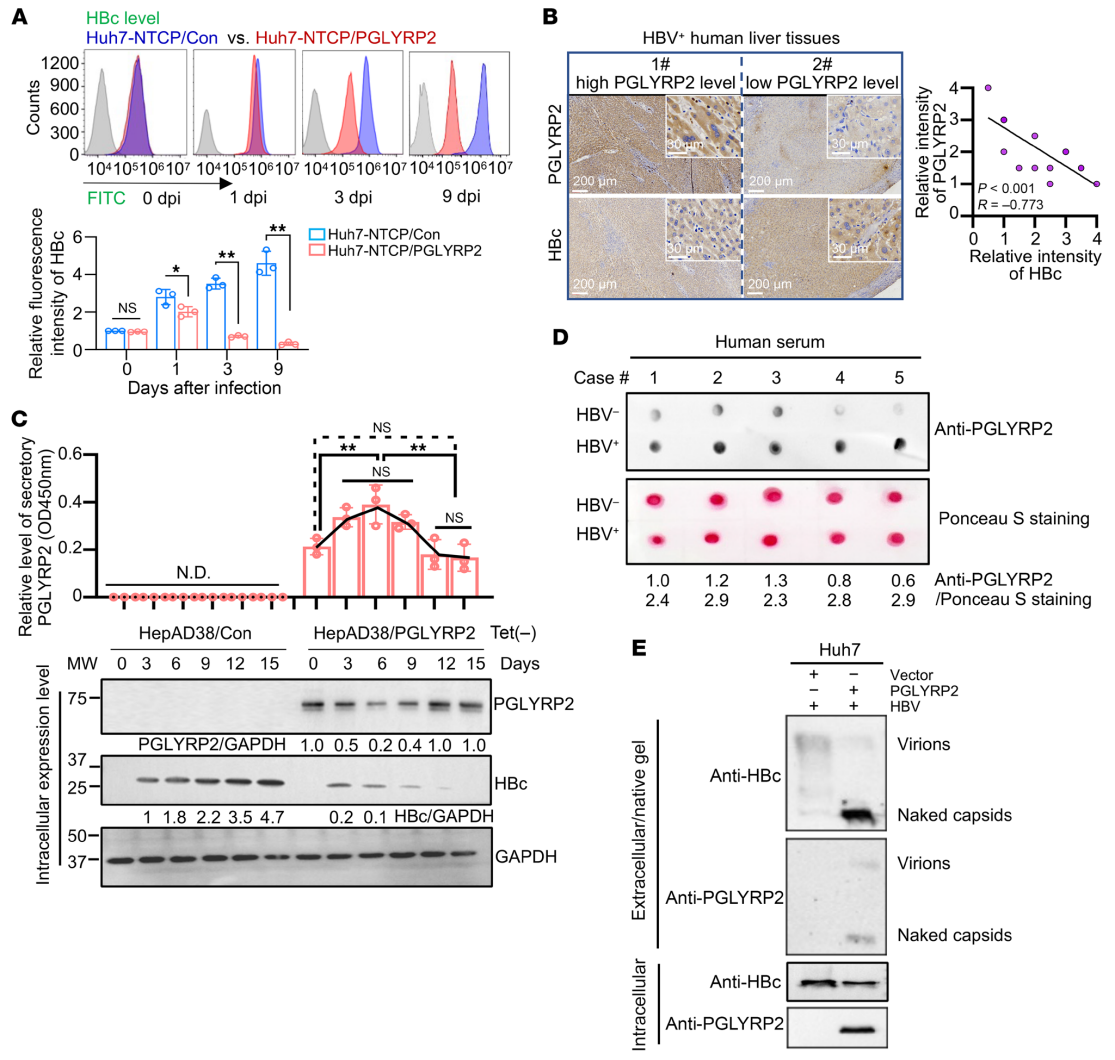
HBV capsid–targeting PGLYRP2 inhibits HBV capsid assembly. (**A**) Top: Flow cytometry was used to assess HBV clearance in Huh7-NTCP/Con and Huh7-NTCP/PGLYRP2 cells infected with HBV at a multiplicity of infection of 1:2,500. Bottom: Quantification of HBc fluorescence intensity. Dots indicate biological replicates (*n* = 3 independent experiments). (**B**) Immunohistochemical staining for PGLYRP2 and HBc in HBV^+^ human liver tissues from distal non-tumor liver tissues of hepatocellular carcinoma patients. The correlation between HBc and PGLYRP2 expression was statistically analyzed. Scale bars: 200 μm. (**C**) Stable cell lines HepAD38/Con and HepAD38/PGLYRP2 were cultured in the medium without Tet for 15 days. Top: Supernatant levels of PGLYRP2 were detected by ELISA. Dots indicate biological replicates (*n* = 3 independent experiments). Bottom: Levels of PGLYRP2 and HBc in the cell lysates were analyzed by Western blot at the indicated times. (**D**) Dot blot analysis was used to measure extracellular PGLYRP2 levels in serum samples from healthy individuals and chronic hepatitis B patients. Each serum sample (10 μL) was applied onto a nitrocellulose membrane, followed by detection using a rabbit anti-PGLYRP2 antibody and an HRP-labeled anti-rabbit IgG. Ponceau S staining served as the loading control, with dot intensities quantified using Multi Gauge software v3.0 (Fujifilm). (**E**) Cotransfection of a PGLYRP2 expression plasmid with an HBV plasmid into Huh7 cells resulted in enhanced secretion of naked capsids. At day 5 after transfection, extracellular HBV particles were isolated by ultracentrifugation and analyzed via 1% native agarose gel electrophoresis, followed by Western blot to assess both capsid and PGLYRP2 levels. Data are represented as mean ± SD. Two-tailed Student’s *t* test (**A**), Pearson’s correlation coefficient (**B**), and 1-way ANOVA with post hoc Bonferroni’s test (**C**) were used for statistical analysis. *P < 0.05; **P < 0.001.

To elucidate the role of HBV-responsive PGLYRP2 translocation in facilitating HBV clearance, stable Huh7-NTCP/Con and Huh7-NTCP/PGLYRP2 cell lines were inoculated with HBV for 48 hours. A flow cytometry–based HBV clearance assay was performed to quantitatively measure HBc levels in HBV-infected cells. At day 0 after infection, there was no significant difference in HBc fluorescence intensity between the two Huh7-NTCP groups, confirming that PGLYRP2 does not affect cell susceptibility to HBV infection. Notably, no HBc fluorescence was detected in NTCP-deficient Huh7/Con/vector and Huh7/PGLYRP2/vector cells, indicating the de novo production of HBV particles in these cells (Supplemental Figure 5A). From day 1 to day 9 after infection, while HBc levels continuously increased in Huh7-NTCP/Con cells, they were significantly reduced in Huh7-NTCP/PGLYRP2 cells, as also observed through confocal microscopy on day 9 after infection (Figure 5A and Supplemental Figure 5B). This reduction confirms the mediating role of PGLYRP2 in HBV clearance within hepatocytes.

Furthermore, the correlation between hepatic PGLYRP2 expression and HBV infection status was examined in HBV^+^ human liver tissues. Lower HBc levels were observed in tissues with high PGLYRP2 expression compared with those with low PGLYRP2 levels, establishing a negative correlation between PGLYRP2 and HBc (*n* = 17, *P* < 0.001; *R* = –0.773, 95% CI: –0.917 to –0.449; Figure 5B and Supplemental Table 3). The dynamic correlation between HBc and PGLYRP2 levels was further investigated in Tet-free medium, where HepAD38/PGLYRP2 cells displayed significantly lower HBc levels compared with HepAD38/Con cells, suggesting an inverse correlation between intracellular PGLYRP2 and HBc levels. Remarkably, PGLYRP2 secretion was enhanced in HepAD38/PGLYRP2 cells expressing HBc, indicating the influence of HBc on PGLYRP2 secretion (Figure 5C). Additionally, endogenous PGLYRP2 levels in the serum of healthy individuals and chronic hepatitis B patients were assessed. Significantly increased secretory levels of PGLYRP2 were found in patients with chronic HBV infection compared with healthy controls (Figure 5D).

To ascertain the physiological relevance of increased PGLYRP2 secretion and its potential role in inhibiting HBV capsid assembly, PGLYRP2 was cotransfected with HBV into Huh7 cells. Extracellular HBV particles were collected on day 5 after transfection via ultracentrifugation and analyzed by 1% native agarose gel electrophoresis. The results demonstrated that PGLYRP2 facilitated the secretion of naked capsids, underscoring its potential as a potent modulator of capsid assembly (Figure 5E).

### Secretion of PGLYRP2/HBV capsid complex reinvigorates antiviral immune response within hepatic microenvironment.

**Figure 6:**
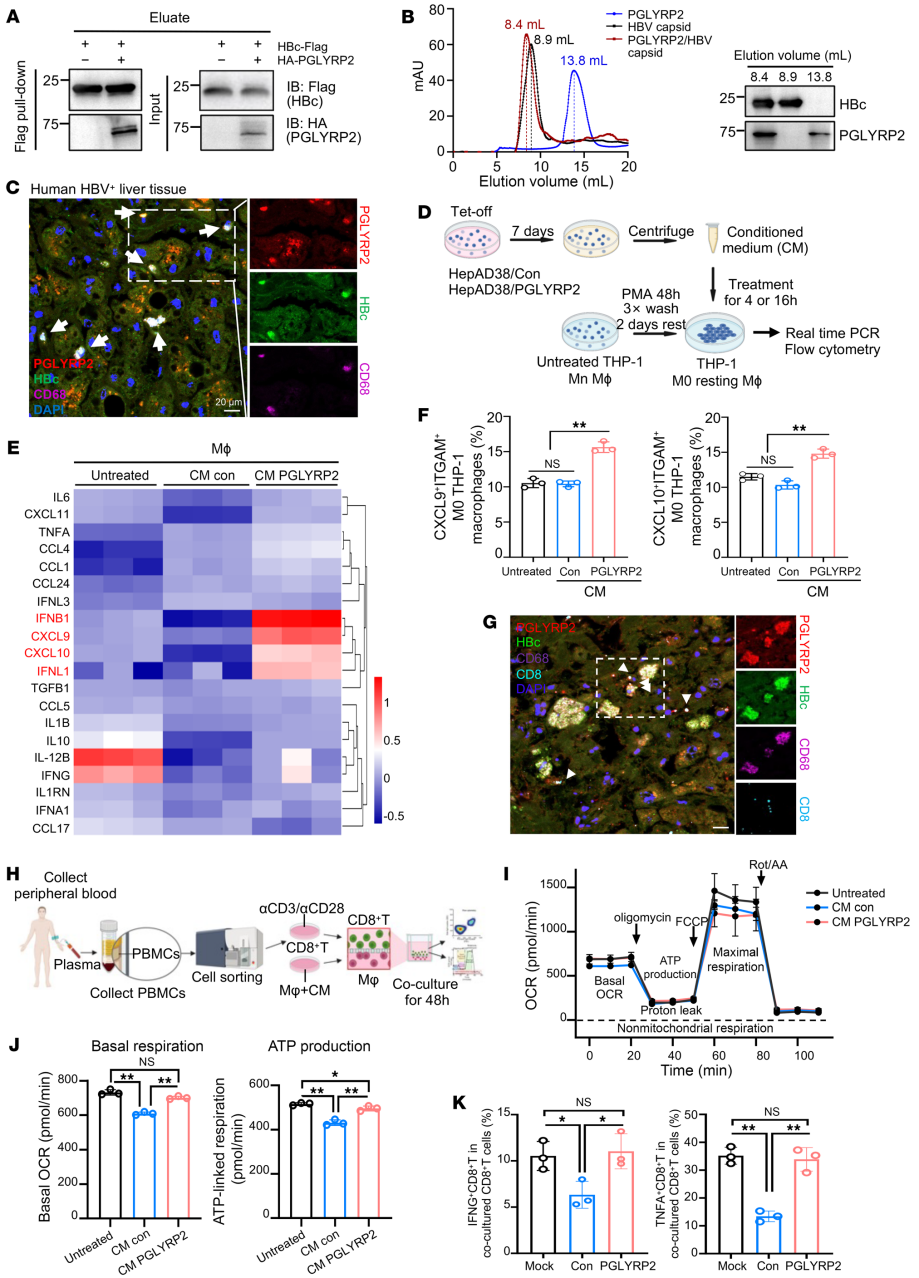
PGLYRP2/HBV capsid complex regulates the functional properties of immune effectors. (**A**) Coimmunoprecipitation analysis to confirm the specificity of interaction. (**B**) Left: Size-exclusion chromatography was performed to discern the molecular complexes formed between PGLYRP2 and HBV capsid. Right: The collected fractions corresponding to elution peaks were further analyzed to validate the protein compositions. (**C**) Multiplex immunofluorescence staining was used to visualize the colocalization of PGLYRP2, HBc, and CD68 in HBV-infected non-tumor liver tissues from HCC patients. (**D**) Conditioned media were prepared from Tet-off HepAD38 cells expressing either control vector or PGLYRP2. THP-1 M0 macrophages were treated with CM for 4 or 16 hours, and changes in the immune profile were assessed. (**E**) Hierarchical clustering analysis revealed distinct cytokine and chemokine profiles in THP-1 M0 macrophages following 4-hour treatment with CM. (**F**) Flow cytometry analysis of THP-1 macrophages treated with CM for 16 hours identified a subset of ITGAM^+^ cells producing CXCL9/10. (**G**) Multiplex immunofluorescence staining in HBV-infected liver tissues corroborated the presence of PGLYRP2, HBc, CD68, and CD8. Scale bar: 20 μm. (**H**) Coculture experiments of CD8^+^ T cells from healthy donors with CM-treated macrophages for 48 hours revealed significant changes in the activation status of these T cells, analyzed by flow cytometry and a Seahorse extracellular flux analyzer. (**I** and **J**) The Seahorse MitoStress Test was conducted to measure the complete oxygen consumption rate (OCR) trace (**I**), basal OCR, and ATP-linked respiration (**J**). (**K**) Intracellular cytokine staining for IFNG and TNFA in cocultured CD8^+^ T cells was performed to evaluate their effector functions. Dots indicate biological replicates (*n* = 3 independent experiments). Data are represented as mean ± SD. One-way ANOVA with post hoc Bonferroni’s test (**F**, **J**, and **K**) was used for statistical analysis. *P < 0.05; **P < 0.001.

To explore whether the PGLYRP2/HBV capsid complex could be released into the extracellular space, cell culture supernatants from HEK293 cells coexpressing HBc and PGLYRP2 were collected. These samples underwent FLAG pull-down assays and ultracentrifugation. Western blot analyses of proteins eluted with 3×FLAG peptides confirmed the formation of an extracellular complex between PGLYRP2 and HBc (Figure 6A). Further purification by size-exclusion chromatography (SEC) revealed a distinct migration pattern for the PGLYRP2/HBV capsid complex compared with the HBV capsid alone, evidenced by a notable peak shift in the SEC elution profile (Figure 6B).

Macrophages, as sentinel cells, play a critical role in sensing pathogens and initiating rapid immune responses. In HBV-infected distal non-tumor liver tissues from hepatocellular carcinoma patients, colocalization of the PGLYRP2/HBV capsid complex with CD68, a pan-macrophage marker, was demonstrated using multiplex immunofluorescence staining, suggesting the presence of the complex within liver macrophages (Figure 6C).

The impact of conditioned medium containing the PGLYRP2/HBV capsid complex (CM-PGLYRP2) on macrophage activation was then assessed. THP-1 monocytes were differentiated into non-activated M0 macrophages using phorbol 12-myristate 13-acetate (PMA) and subsequently treated with the conditioned medium. Real-time PCR and flow cytometry revealed that CM-PGLYRP2 significantly increased the production of proinflammatory cytokines and chemokines, such as IFNB1, IFNL1, and CXCL9/10, compared with untreated or CM-Con–treated M0 macrophages, confirming the immunostimulatory effect of CM-PGLYRP2 (Figure 6, D–F, and Supplemental Figure 6, A and B). This enhancement was not observed in HepG2-derived CM-treated Raw264.7 macrophages, indicating that the regulatory effect of PGLYRP2 is specific to HBV-infected conditions (Supplemental Figure 6C). Additionally, our findings show that M1 macrophages exposed to CM-PGLYRP2 produce significantly higher levels of proinflammatory cytokines such as IL-6 and TNFA compared with those treated with CM-Con (Supplemental Figure 6, D and E).

Furthermore, multiplexed immunofluorescence revealed occasional proximity of CD8^+^ T cells to macrophages harboring the PGLYRP2/capsid complex (Figure 6G and Supplemental Figure 6D). Considering that fine-tuning of mitochondrial respiration is important for the antiviral activity of CD8^+^ T cells (47, 48), the functional impact on CD8^+^ T cells was assessed by measurement of mitochondrial function using a Seahorse assay and flow cytometry (Figure 6H). Results indicated a decrease in basal respiration in CD8^+^ T cells cocultured with CM-Con–treated macrophages, whereas maximal respiration and proton leak remained unchanged across different treatment groups (Figure 6I and Supplemental Figure 6E). Additionally, ATP production was significantly reduced in CD8^+^ T cells cocultured with CM-Con–treated macrophages, contrasting with elevated IFNG and TNFA production in cells cocultured with CM-PGLYRP2–treated macrophages (Figure 6, J and K, and Supplemental Figure 6H). These findings underscore the pivotal role of the PGLYRP2/HBV capsid complex in enhancing the functional properties of macrophages and CD8^+^ T cells within the viral microenvironment, thereby bolstering the antiviral immune response.

### Compromised HBV control in PGLYRP2-deficient hepatocytes and the target specificity of human PGLYRP2.

**Figure 7:**
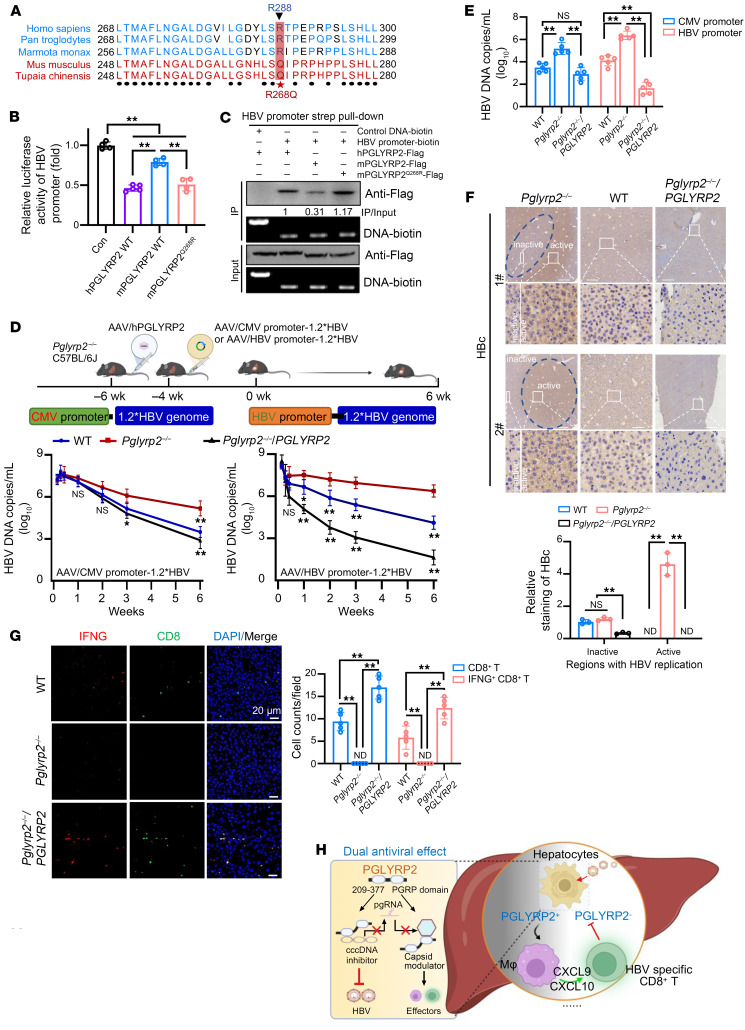
*PGLYRP2* deficiency compromises HBV control in hepatocytes. (**A**) Multiple sequence alignment of PGLYRP2 proteins from various species. (**B**) Cotransfection of HBV promoter–luciferase reporter constructs with human PGLYRP2 (hPGLYRP2), mouse PGLYRP2 (mPGLYRP2), or mutant mPGLYRP2^Q268R^ into C3A cells. Renilla luciferase was used as a control for transfection efficiency. Dots indicate biological replicates (*n* = 4 for Con, mPGLYRP2 WT, and mPGLYRP2^Q268R^, *n* = 5 for hPGLYRP2 WT; independent experiments). (**C**) Purification of HBV promoter–binding proteins (hPGLYRP2, mPGLYRP2, mPGLYRP2^Q268R^) via DNA pull-down assays followed by Western blot and agarose gel analysis. (**D**) *Pglyrp2-*WT, *Pglyrp2-*knockout (*Pglyrp2^–/–^*), and *Pglyrp2*-knockout replaced with human *PGLYRP2* (*Pglyrp2^–/–^*/*PGLYRP2*) C57BL/6J mice (*n* = 8) were injected with pAAV vectors expressing HBV. The vectors used were pAAV-CMV/HBV1.2 (left) or pAAV-HBV core promoter/HBV1.2 (right). Serum HBV titers were quantitatively measured by real-time PCR at indicated times after injection. (**E**) Comparison of HBV serum titers in WT, *Pglyrp2^–/–^*, and *Pglyrp2^–/–^*/*PGLYRP2* mice at 10 weeks after injection. Dots indicate biological replicates (*n* = 5 independent experiments). (**F**) Immunohistochemical analysis of HBc expression in mouse liver sections after injection with pAAV-HBV promoter/HBV1.2, complemented by quantitative evaluation of HBc staining in regions active or inactive in HBV replication. Dots indicate biological replicates (*n* = 3 independent experiments). (**G**) Left: Immunofluorescence staining for IFNG (red) and CD8 (green) in HBV-infected WT, *Pglyrp2^–/–^*, or *Pglyrp2^–/–^*/*PGLYRP2* liver tissues from HBV mouse model at 10 weeks after AAV-HBV promoter/1.2×HBV injection. Right: Statistical analysis of CD8^+^ T and IFNG^+^ CD8^+^ T cell counts in HBV-infected mouse liver tissues. Dots indicate biological replicates (*n* = 3 independent experiments). (**H**) Schematic depiction of PGLYRP2-mediated HBV clearance. Data are represented as mean ± SD. One-way ANOVA with post hoc Bonferroni’s test (**B** and **E**–**G**) and 2-way ANOVA with post hoc Bonferroni’s test (**D**) were used for statistical analysis. *P < 0.05; **P < 0.001.

During a multiple sequence alignment of 5 representative PGLYRP2 proteins across various species, a missense single-nucleotide polymorphism (SNP), an R-to-Q change at position 268 (R268Q), was identified in the mouse PGLYRP2 protein (Figure 7A). Subsequent analyses suggested that this SNP could potentially impact the suppression of HBV replication. We used an HBV promoter–luciferase reporter assay to evaluate the effect of this mutation on HBV suppression. The results demonstrated that the inhibition of HBV replication by mouse PGLYRP2 (mPGLYRP2) was significantly less effective in comparison with human PGLYRP2 (hPGLYRP2). Conversely, the Q268R mutation in mPGLYRP2 enhanced the suppressive effect on HBV replication (Figure 7B). A biotin-streptavidin affinity pull-down assay confirmed that, relative to mPGLYRP2, both hPGLYRP2 and mPGLYRP2^Q268R^ exhibited stronger HBV DNA binding capabilities (Figure 7C and Supplemental Figure 7A).

To determine whether PGLYRP2-mediated immune responses were linked to HBV clearance in mouse models, adeno-associated virus (AAV) vectors carrying HBV1.2 under CMV or HBV promoters were administered to WT, *Pglyrp2^–/–^*, and *Pglyrp2^–/–^*/*PGLYRP2* C57BL/6J mice (for *Pglyrp2^–/–^*/*PGLYRP2* C57BL/6J mice, AAV/hPGLYRP2 vectors were i.v. injected into the tail vein). Periodic blood sampling monitored HBV DNA levels in the serum. In *Pglyrp2^–/–^* mice, the decline in HBV DNA levels was significantly slower compared with that in WT and *Pglyrp2^–/–^*/*PGLYRP2* mice, indicating a crucial role for PGLYRP2 in HBV control. Notably, HBV DNA levels in *Pglyrp2^–/–^*/*PGLYRP2* mice receiving the pAAV-HBV promoter/HBV1.2 were considerably lower than those in their WT counterparts, highlighting the target specificity of hPGLYRP2 (*P* < 0.001; Figure 7, D and E).

To further investigate the impact of PGLYRP2 on HBV replication and clearance, liver tissues and serum from HBV mouse models were analyzed at 10 weeks after injection with AAV-HBV promoter/1.2×HBV. Strong HBc staining, indicative of active viral replication, was observed exclusively in liver sections from *Pglyrp2^–/–^* mice infected with HBV. In contrast, weak HBc staining was noted in liver sections from *Pglyrp2^–/–^*/*PGLYRP2* mice; moderate staining was observed in liver sections of WT mice and in the inactive regions of HBV replication in *Pglyrp2^–/–^* mice (Figure 7F and Supplemental Figure 7B). The suppression of HBs and HBe production in the serum of the mouse model, along with the marked reduction in intrahepatic pgRNA levels, further corroborated the involvement of hPGLYRP2 in viral clearance, with serum alanine aminotransferase (ALT) levels remaining within the physiological range (Supplemental Figure 7, C and D).

The activation status of CD8^+^ T cells in the liver tissues was investigated using an immunofluorescence assay. No CD8^+^ T cells or IFNG^+^ CD8^+^ T cells were detectable in the liver tissues of HBV-infected *Pglyrp2^–/–^* mice. In contrast, moderate to high cell counts of CD8^+^ T cells and IFNG^+^ CD8^+^ T cells were identified in the liver tissues of infected WT or *Pglyrp2^–/–^*/*PGLYRP2* mice, respectively (Figure 7G). These findings demonstrate that PGLYRP2 plays a critical role in HBV control and clearance, with hPGLYRP2 exhibiting target specificity in its interaction with HBV. The compromised HBV control in *PGLYRP2*-deficient hepatocytes highlights the importance of PGLYRP2 in the immune response against HBV infection.

## Discussion

Recent paradigm shifts in research on HBV challenge the former notion of it as a “stealth” virus, highlighting the critical role of host antiviral immune responses in initially suppressing the virus. While the mechanisms driving effective innate immune responses and spontaneous viral clearance are not fully elucidated, our study underscores the importance of the interplay between host innate immunity and HBV in developing therapeutic strategies. Using a pull-down assay, we identified previously reported HBV-related proteins (DDB2, YBX1, HMGB1) alongside potential HBV promoter–binding proteins, including PGLYRP2, which may regulate HBV transcription. Our results demonstrate that hepatic PGLYRP2 enhances age-dependent HBV clearance through both direct antiviral actions and immunomodulatory effects, positioning PGLYRP2 as a promising candidate for anti-HBV drug development aimed at targeting viral cccDNA and capsid assembly (Figure 3G and Figure 4G). Furthermore, in HBV-infected hepatocytes, the induction of interferons and interferon-stimulated genes (ISGs) is relatively weak. However, hepatocytes stably expressing PGLYRP2 exhibit markedly enhanced induction of interferons and ISGs, underscoring the role of PGLYRP2 in promoting hepatocyte-intrinsic innate immunity (Supplemental Figure 7E). These findings deepen the understanding of the mechanisms underlying HBV infection and replication and illuminate the complex network of host cell responses to HBV invasion. Additionally, further investigation of the HBV-associated protein PGLYRP2 in this study may provide a more comprehensive understanding of the host response strategy to HBV infection, potentially leading to the identification of antiviral strategies.

The critical role of host recognition of HBV DNA and antigens in viral clearance is well documented (49, 50). Our findings expand on this understanding by showing that PGLYRP2 not only suppresses HBV replication and cccDNA formation through interactions with HBV DNA but also possesses the ability to bind to the HBV capsid, thereby amplifying its antiviral efficacy. An increase in HBV load, which escalates capsid levels, promotes the nuclear-cytoplasmic translocation of PGLYRP2. Predictions from the AlphaFold3 structural model suggest that the nuclear localization signal (NLS) at the C-terminus, adjacent to the PGRP domain, might be obscured by steric hindrance from the interaction with the HBV capsid. This obstruction necessitates the role of the signal peptide (SP) at the N-terminus in facilitating the virus-responsive translocation of the molecule. This dynamic shuttling of PGLYRP2 underscores its vital role in spontaneous HBV clearance and emphasizes its function as a surveillance molecule in ongoing immune defense.

Moreover, the age-dependent expression pattern of *PGLYRP2* observed in this study may contribute to the differing susceptibilities to HBV infection between adults and children. The presence of PGLYRP2 SNPs in different populations, particularly within the PGLYRP2^209–377^ domain (such as the SNPs T257N, M270K, R288Q, and S297C described here), appears to influence the anti-HBV activities and could potentially explain the variation in outcomes observed in approximately 5% of adults who develop chronic HBV infections. Additionally, scRNA-Seq data have revealed substantial heterogeneity in hepatocyte *PGLYRP2* expression, which is positively correlated with *NTCP* levels and inversely related to HBV infection status across the liver (Supplemental Figure 8). This heterogeneity may account for the variable anti-HBV abilities of hepatocytes and could provide insight into why chronic HBV infections persist in some patients with lower *PGLYRP2* expression in their hepatocytes (51, 52).

In conclusion, our findings affirm the pivotal role of PGLYRP2 in innate antiviral immunity, mediated through its dual regulatory effects on HBV suppression and modulation of the hepatic microenvironment during infection. Exploiting the dual-functional domains of PGLYRP2 — the direct-acting antiviral domain (PGLYRP2^209–377^, a cccDNA inhibitor) and the immunomodulatory domain (PGLYRP2^PGRP^, a capsid assembly modulator) — presents a promising therapeutic pathway (Figure 7H). Considering the limitations of current therapies in completely eradicating chronic HBV infection, a combination therapy involving distinct domains of PGLYRP2 holds promise for a comprehensive and effective cure.

## Methods

### Sex as a biological variable.

Our study included both male and female patients and mice, and similar findings were observed. The results are expected to be relevant regardless of biological sex.

### Mouse strain and tissue specimens.

Wild-type (WT) and *Pglyrp2^–/–^* C57BL/6J mice were sourced from Cyagen Biosciences, Guangzhou. For experimental procedures, pAAV/hPGLYRP2, pAAV-CMV/HBV1.2, or pAAV-HBV promoter/HBV1.2 vectors (5 × 10^11^ viral genomes diluted in 200 μL PBS) were administered via tail vein injection to C57BL/6J mice. All mice were housed under standard laboratory conditions, maintained at 22°C with a 12-hour light/12-hour dark cycle, and provided with food and water ad libitum. Housing was in micro-isolator cages equipped with wire racks for food and water bottles.

Human liver tissue specimens were collected from distal non-tumor regions of the livers of 19 liver cancer patients. Informed consent was secured from the relatives of all study participants.

### Cell lines and plasmid construction.

Huh7, HEK293T, and THP-1 cells were originally purchased from ATCC. C3A cells were from the cell bank of Type Culture Collection of Chinese Academy of Sciences. Huh7, C3A, and HEK293T cells were cultured in DMEM supplemented with 10% fetal bovine serum (FBS; ScienceCell). THP-1 cells were cultured in RPMI 1640 medium supplemented with 10% FBS. CD8^+^ T cells from PBMCs were cultured in RPMI 1640 medium supplemented with 10% FBS. HepAD38 cells were cultured in DMEM/F12 medium supplemented with 10% FBS and 400 μg/mL G418 and 0.3 μg/mL tetracycline. All the cells were cultured in a humidified incubator with 5% CO_2_ at 37°C and were tested for mycoplasma contaminations.

Stable cell lines Huh7-NTCP/Con, Huh7-NTCP/PGLYRP2, HepAD38/Con, and HepAD38/PGLYRP2 were generated using a lentiviral system. Lentiviral particles were produced in HEK293T cells by cotransfection with 5 μg of the lentiviral expression plasmid pLV-CMV/target gene, 4.5 μg of the packaging plasmid pCMV-GAG, and 500 ng of the envelope-expressing plasmid pCMV-VSV-G. After transfection, the culture medium was replaced after 24 hours with fresh DMEM supplemented with 10% FBS, and viral supernatants were harvested at 72 hours. Approximately 4 × 10^5^ Huh7 or HepAD38 cells were transduced with these lentiviral particles in the presence of 10 μg/mL Polybrene (Biosharp) and cultured for 48 hours. Stable monoclonal lines were selected using puromycin for 7 days and validated by Western blot analysis.

The 3×FLAG tag was integrated into the pLVSIN-CMV-puro vector at the *Not*I/*Bam*HI restriction sites. For the construction of eukaryotic expression plasmids for HBc, the coding region of HBc was cloned into the pCMV-HA vector at the *Eco*RI/*Kpn*I sites, and into the pLVSIN-CMV-puro-3×FLAG vector at the *Xho*I/*Not*I sites. The HA tag or FLAG tag was fused to either the N-terminus or the C-terminus of HBc, respectively.

Primary human hepatocytes (PHHs) were isolated from excess liver tissue obtained during surgical liver tumor resections, using a modified 2-step collagenase perfusion technique (53). The donor characteristics and relevant details are documented in Supplemental Table 4. Once isolated, PHHs were plated on rat tail collagen–coated cell culture dishes (BD) and cultured in HepatoZYME-SFM medium (Gibco), ensuring optimal conditions for cellular maintenance and experimental reproducibility.

### Immunofluorescence and multiplex immunoassays.

Immunofluorescence staining was performed as described previously (54). Tissue sections were observed under a Zeiss Axioskop-2 microscope, using 488-nm- and 633-nm-wavelength lasers for the excitation of fluorescein tags, with DAPI excited by UV light to visualize nuclear structures.

Human liver tissue specimens underwent multiplex immunofluorescence using the Novo Light TSA 5 colors kit (WiSee Biotech, Shanghai) according to the manufacturer’s protocols. Paraffin-embedded sections were first deparaffinized, followed by heat-induced epitope retrieval in sodium citrate solution. The multiplex staining involved primary antibodies targeting CD8 (A11856, ABclonal), CD68 (ab955, Abcam), PGLYRP2 (NBP2-32042, Novus Biologicals), and HBc (NB110-7396, Novus). After application of secondary antibodies conjugated with HRP (ZSGB-BIO), tyramide signal amplification–conjugated (TSA-conjugated) fluorophores were used to enhance signal intensity and specificity, with DAPI serving as the nuclear counterstain. Imaging of the stained tissue slices was performed using the Vectra 3.0 spectral imaging system (PerkinElmer), enabling detailed visualization and analysis of the biomarkers.

### Measurement of oxygen consumption rate and Seahorse XF analysis.

PBMC-derived CD8^+^ T cells were seeded into an XFe24 cell culture plate (Agilent Technologies) to achieve optimal confluence for the assay. After a 48-hour incubation with macrophages pretreated with CM from HepAD38 Tet-off cells in a Transwell system, mitochondrial respiration was assessed using the XFe24 Extracellular Flux Analyzer (Seahorse Bioscience, Agilent Technologies). Before the measurements, each well of the utility plate was prepared with 1 mL of XF Calibrant fluid, and the sensor plate was equilibrated overnight at 37°C in an incubator. On the day of the assay, cells were transitioned from their culture medium to a specially formulated assay medium and allowed to equilibrate for an additional hour at 37°C before initiation of baseline respiration measurements.

After baseline assessment, a series of mitochondrial inhibitors — oligomycin (an ATP synthase inhibitor), FCCP (a mitochondrial uncoupler), and a combination of rotenone and antimycin A (complex I and III inhibitors, respectively) — were sequentially injected into each well. These chemicals were prepared in the assay medium to precisely measure changes in the oxygen consumption rate, which was recorded in picomoles per minute. All consumables and reagents used during this procedure were sourced from Seahorse Bioscience (Agilent Technologies), ensuring high-quality and standardized conditions for reproducibility of the results.

### Proximity ligation assay.

The proximity ligation assay (PLA) was conducted using Duolink In Situ PLA Probe Anti-Rabbit PLUS (DUO92002, Sigma-Aldrich) and Anti-Mouse MINUS (DUO92004, Sigma-Aldrich), along with Duolink In Situ Detection Reagents Red (DUO92008, Sigma-Aldrich), strictly following the manufacturer’s guidelines. All procedures were carried out in a humidity-controlled chamber to prevent sample drying. The assay began with an overnight incubation at 4°C, where mouse anti-HBc antibody (NB110-7396, Novus) and rabbit anti-PGLYRP2 antibody (NBP2-32042, Novus) were applied to the samples, followed by the Streptavidin PLA-PLUS probe (Merck).

After primary antibody binding, a donkey anti-rabbit antibody conjugated with Alexa Fluor 488 was used to detect the rabbit anti-PGLYRP2 antibody. After PLA processing, coverslips were thoroughly rinsed with MilliQ H_2_O and then mounted on glass slides using Fluoromount (Sigma-Aldrich). Images of the samples were acquired and analyzed using ImageJ software (NIH), focusing on estimating the percentage of PLA- and PGLYRP2-double-positive spots relative to the total PGLYRP2-positive cells.

### ELISA, cell sorting, and FACS analysis.

The secretion of HBeAg and HBsAg was quantified using a specific ELISA kit (Cyttel, Beijing). Cell sorting and fluorescence-activated cell sorting (FACS) analyses were performed. For the isolation of CD8^+^ T cells from PBMCs, cells were stained using fluorescently conjugated antibodies: CD3-APC (OKT3, BioLegend) and CD8-FITC (RPA-T8, BioLegend). The CD3^+^CD8^+^ T cells were subsequently sorted using a FACSAria II Cell Sorter (BD Biosciences).

The levels of HBc in HBV-infected Huh7-NTCP cells and the functional properties of various immune cell subtypes were analyzed via FACS. Cells were labeled with a panel of fluorescence-conjugated monoclonal antibodies and sorted using a BD Accuri C6 Plus Personal Flow Cytometer (BD Biosciences). The specific sorting criteria included M0 THP-1 and CD68^+^ macrophages stained with CD68-PE (Y1/82A, BioLegend), ITGAM-FITC (ICRF44, BioLegend), CXCL9-APC (J1015E10, BioLegend), and CXCL10-APC (J034D6, BioLegend). CD8^+^ T cells were identified using CD8-FITC (RPA-T8, BioLegend), IFNG-PE (4S.B3, BioLegend), and TNFA-APC (Mab11, BioLegend). HBV^+^ Huh7-NTCP cells were detected with a primary antibody specific for HBc (orb99015, Biorbyt) and a secondary goat anti-mouse antibody conjugated with Alexa Fluor 488 (Invitrogen). For intracellular cytokine staining, approximately 10^6^ cells per sample were cultured in RPMI medium containing 2% FBS and stimulated for 4 hours with PMA (50 ng/mL; Sigma-Aldrich), ionomycin (2 μg/mL; Sigma-Aldrich), and Golgiplug (1.5 μL/mL; Thermo Fisher Scientific). Cells were then fixed, permeabilized, and stained with cytokine-specific antibodies. Data analysis was performed using FlowJo software (version 10.8.1), facilitating detailed evaluation of cellular phenotypes and their functional attributes in the context of HBV infection.

### Quantitative real-time PCR and luciferase reporter assays.

Total RNA was isolated from samples using the Trizol reagent (Ambion) according to the manufacturer’s protocol. The extracted RNA was then reverse-transcribed to cDNA using the PrimeScript RT Reagent Kit with gDNA Eraser (Takara), ensuring the removal of genomic DNA and enhancing the purity of the RNA template. The amplification of cDNA was performed using the ABI 7500 Real-Time PCR System (Applied Biosystems). SYBR Green PCR Master Mix (Cowin Bio) was used to enable the detection of PCR products in real time. Each reaction was conducted under optimal conditions to ensure high specificity and efficiency. The quantitative reverse transcription PCR results were validated through at least 3 independent experiments, confirming the reproducibility and reliability of the data.

Luciferase assays were integral for evaluating promoter activity within the cellular context. Transfected cells were analyzed for luciferase activity to assess the transcriptional regulation of target genes. HBV promoter activity was analyzed using a Dual-Glo Luciferase Assay System (Promega), and luminescence was measured using a Turner Biosystems instrument.

### Immunoblotting and immunoprecipitation.

For immunoblotting, cells were lysed using RIPA buffer (50 mM Tris-HCl [pH 7.4], 150 mM NaCl, 1% NP-40, 0.5% sodium deoxycholate), which was supplemented with a protease inhibitor cocktail (Biosharp) to prevent protein degradation. Lysates were then centrifuged to remove insoluble material, and the supernatants were subjected to separation by 12% SDS-PAGE. Proteins were subsequently transferred onto PVDF membranes for analysis. For detection, membranes were incubated with specific primary antibodies overnight at 4°C, followed by appropriate HRP-conjugated secondary antibodies for 1 hour at room temperature. Protein bands were visualized using enhanced chemiluminescence (ECL) reagent (Thermo Fisher Scientific).

For immunoprecipitation, cell lysates were incubated with target-specific antibodies and agarose beads at 4°C overnight to form immunocomplexes. After incubation, beads were washed thoroughly with lysis buffer to remove non-specifically bound proteins. The bound immunocomplexes were then eluted and analyzed via immunoblotting, using the same antibody detection methodology as described for the immunoblotting procedure.

### Immunohistochemistry.

The immunohistochemical staining was performed as described previously (23). Antibodies against human PGLYRP2 (NBP2-32042, Novus) and mouse PGLYRP2 (MAB4704, R&D Systems) were purchased from Novus Biologicals. Antibody against HBc (orb99015) was from Biorbyt.

### Chromatin immunoprecipitation.

ChIP assays were conducted to examine the interaction between PGLYRP2 and the HBV promoter, using the ab500 ChIP kit (Abcam). Cells were cross-linked with 1% formaldehyde, followed by neutralization with 125 mM glycine. The lysates were sonicated, resulting in DNA fragments between 200 bp and 500 bp. Immunoprecipitation was performed using Anti-FLAG Magnetic Agarose (A36797, Thermo Fisher Scientific). DNA was purified and amplified by PCR using specific primers. The amplified samples were then subjected to PCR analysis and agarose gel electrophoresis to confirm the presence of target sequences.

### Biotin-streptavidin affinity pull-down assay.

The biotin-streptavidin affinity pull-down assay was used to analyze protein-DNA interactions. Biotin-labeled HBV promoter DNA fragments were first amplified and purified using the Gel Extraction Kit (Thermo Fisher Scientific). Streptavidin magnetic beads (MedChemExpress) were incubated with the purified DNA fragments for 1 hour at room temperature. Subsequently, these beads, now conjugated with biotin-labeled DNA, were incubated with cell lysates for 4 hours at 4°C. Proteins of interest bound to the magnetic beads were then analyzed by liquid chromatography–tandem mass spectrometry and Western blot to identify and quantify bound components.

### Electrophoretic mobility shift assay.

EMSA was used to assess the cccDNA binding activity of PGLYRP2. Binding reactions were set up by incubation of 300 ng of MfeI-digested cccDNA with either 100 pmol of purified PGLYRP2 proteins or 3×FLAG peptides. The reactions took place in 0.5× TBE buffer, which included 150 mM NaCl, at 4°C for 60 minutes. After incubation, the products of the binding reactions were separated on a native 1% agarose gel. The complexes were subsequently analyzed by both Southern blotting and Western blotting to confirm the specificity and integrity of the protein-DNA interactions.

### cccDNA detection and quantification.

HBV cccDNA was isolated from HepAD38/Con and HepAD38/PGLYRP2 cells cultured in Tet-free medium using the Hirt extraction method. The isolated cccDNA was subjected to enzymatic treatment involving ExoI, ExoIII, and T5 exonuclease. Specifically, the cccDNA was treated with 5 U of ExoI and 25 U of ExoIII for 2 hours at 37°C. After this, the reaction mixture was extracted with phenol-chloroform, and the DNA was precipitated with ethanol and resuspended in 20 μL of Tris-EDTA (TE) solution. This eluent was further treated with 5 U of T5 exonuclease for an additional hour. After another round of phenol-chloroform extraction, the purified cccDNA, resistant to exonuclease digestion, was dissolved in 20 μL TE. Quantification of HBV cccDNA was performed using real-time quantitative PCR. The specific primers used are detailed in Supplemental Table 5.

For robust detection, the purified HBV cccDNA underwent MfeI digestion to linearize it into dsDNA, which was then resolved on a 1% (wt/vol) agarose gel. The DNA within the gel was transferred onto a positively charged nylon membrane (Sigma-Aldrich) using 20× SSC buffer. The DNA was cross-linked to the membrane using UV irradiation at 120,000 μJ/cm^2^ and prehybridized using the solution from the DIG-High Prime DNA Labeling and Detection Starter Kit II (Roche) for 1 hour at 42°C. Hybridization with digoxin-labeled (DIG-labeled) HBV probes occurred at 42°C for 14 hours. Detection was achieved with a chemiluminescence imaging system (SH-523, Shenhua), and the cccDNA bands were quantified using ImageJ software.

### Fluorescence recovery after photobleaching.

The fluorescence recovery after photobleaching (FRAP) measurement of HBV DNA FAM–Enh II within PGLYRP2^IDR/209–377^-induced condensates was conducted using a Nikon A1 laser scanning confocal microscope equipped with a ×60/1.2 NA water immersion objective. For imaging, a 488 nm argon laser at 0.5% power was used, and photobleaching was performed with 1 iteration at 50% laser power. The scanning parameters encompassed a frame size of 256 × 256 pixels, captured at a frequency of 4 frames per second over a duration of 60 seconds after bleaching, with a pixel size of 0.12 μm. A specific circular region of 2.9 μm in diameter was targeted for bleaching with a single 60-millisecond duration using a high-intensity 50% laser. Twenty-five pre-bleach images were captured, and the fluorescence intensity of the last 10 was averaged to normalize the post-bleach fluorescence recovery curve. Each experimental condition involved FRAP analysis on at least 10 condensates and was replicated at least twice to ensure reliability.

### Statistics.

Statistical analysis was performed using GraphPad Prism 10 software. Data are expressed as mean ± SD. Comparisons between 2 groups were conducted using 2-tailed unpaired Student’s *t* test or Mann-Whitney test, as appropriate. For comparisons among multiple groups, 1-way ANOVA with post hoc Bonferroni’s test or 2-way ANOVA with post hoc Bonferroni’s test was used, as indicated in each figure legend. *P* values ≤ 0.05 were considered statistically significant; *P < 0.05, **P < 0.001.

### Study approval.

All animal procedures were conducted in compliance with ethical standards approved by the Institutional Animal Care and Use Committee of Harbin Medical University Cancer Hospital (approval IACUC-2021001).

Ethical approval for the use of human tissues in research was granted by the ethics committee of Harbin Medical University Cancer Hospital (approval HMUCH-2021-02). All subjects provided written informed consent prior to participation.

### Data availability.

Values for all data points in graphs are reported in the Supporting Data Values file. The RNA-Seq data (GSE148790 infant liver data, GSE183915 adult liver data, GSE61279 liver data) and scRNA-Seq liver data (GSE124395) are accessible in the NCBI’s Gene Expression Omnibus (GEO) database, along with Human Protein Atlas database RNA-Seq normal tissues (PRJEB4337) and Liver Hepatocellular Carcinoma (TCGA, Pan-Cancer Atlas).

## Author contributions

YL, HM, YY, HW, YZ, TH, HR, BL, and MS were pivotal in designing and conducting experiments, analyzing the data, creating figures, and drafting the manuscript. HR, YQ, JF, JS, YW, HZ, and YZ contributed to the establishment of the mouse model, collection of experimental samples, and data interpretation. YZ, JH, YY, HL, and MS played crucial roles in data interpretation, manuscript writing, and revisions. YY, HW, and MS provided financial support. MS also conceptualized and supervised the study, further analyzing data. The order of co–first authors was determined by the duration of their contributions to the project.

## Supplementary Material

Supplemental data

Unedited blot and gel images

Supplemental video 1

Supporting data values

## Version 1 02/13/2025

In-Press Preview

## Version 2 04/15/2025

Electronic publication
