# Supplementary material for: PGLYRP2 drives hepatocyte-intrinsic innate immunity by trapping and clearing hepatitis B virus
Source: J Clin Invest. 2025 Feb 13;135(8):e188083. doi: 10.1172/JCI188083 (PMC11996887; doi:10.1172/JCI188083)
Supplement: Supplemental data [file jci-135-188083-s222.pdf]

Supplementary Materials for

# PGLYRP2 Drives Hepatocyte-Intrinsic Innate Immunity by Trapping and Clearing Hepatitis B Virus

Ying Li[1,2†], Huihui Ma[1,3†], Yongjian Zhang[4†], Tinghui He[1†], Binyang Li[1,3†], Haoran Ren[1,3†], Jia Feng[1], Jie Sheng[1], Kai Li[5], Yu Qian[1], Yunfeng Wang[6], Haoran Zhao[6], Jie He[1], Huicheng Li[3,7], Hongjin Wu[2,8*], Yuanfei Yao[9*] and Ming Shi[1,3*]

Corresponding author: Yuanfei Yao, Hongjin Wu and Ming Shi (Lead).

E-mail: yaoyuan.fei@hmu.edu.cn; wuhongjin@hit.edu.cn; shiming@hit.edu.cn.

The file includes:

Figs. S1 to S8

Tables S1 to S4

Table of contents

Supplementary figures..........................................................................................................1

Supplementary Tables..........................................................................................................16

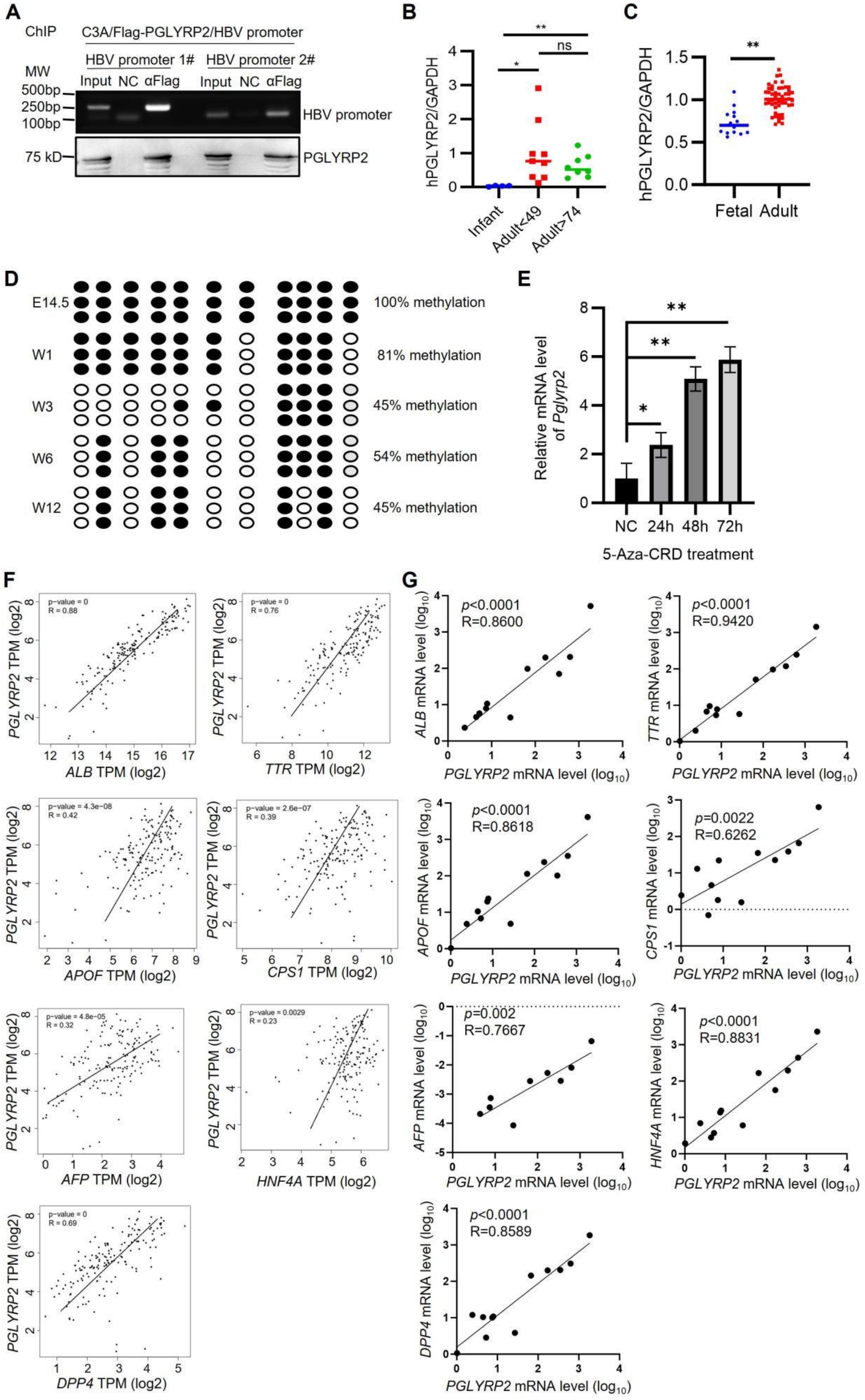
A
ChIP
C3A/Flag-PGLYRP2/HBV promoter
HBV promoter 1#
HBV promoter 2#
MW
Input NC αFlag Input NC αFlag
500bp
250bp
100bp
HBV promoter
75 kD
PGLYRP2
B
hPGLYRP2/GAPDH
Infant
Adult<49
Adult>74
ns
C
hPGLYRP2/GAPDH
Fetal Adult
D
E14.5
100% methylation
W1
81% methylation
W3
45% methylation
W6
54% methylation
W12
45% methylation
E
Relative mRNA level of Pglyrp2
NC 24h 48h 72h
5-Aza-CRD treatment
F
p-value = 0
R = 0.88
PGLYRP2 TPM (log2)
ALB TPM (log2)
p-value = 0
R = 0.76
TTR TPM (log2)
p-value = 4.3e-08
R = 0.42
APOF TPM (log2)
p-value = 2.6e-07
R = 0.39
CPS1 TPM (log2)
p-value = 4.8e-05
R = 0.32
AFP TPM (log2)
p-value = 0.0029
R = 0.23
HNF4A TPM (log2)
p-value = 0
R = 0.69
DPP4 TPM (log2)
G
p<0.0001
R=0.8600
ALB mRNA level (log10)
PGLYRP2 mRNA level (log10)
p<0.0001
R=0.9420
TTR mRNA level (log10)
p<0.0001
R=0.8618
APOF mRNA level (log10)
p=0.0022
R=0.6262
CPS1 mRNA level (log10)
p=0.002
R=0.7667
AFP mRNA level (log10)
p<0.0001
R=0.8831
HNF4A mRNA level (log10)
p<0.0001
R=0.8589
DPP4 mRNA level (log10)

**Fig. S1. Regulatory mechanisms that govern the age-dependent expression pattern of *PGLYRP2*, related to Fig. 1.** A, ChIP assay was employed to explore the interaction between PGLYRP2 and the HBV promoter. C3A cells were co-transfected with a PGLYRP2 expression plasmid and a 1.3xHBV promoter-containing plasmid for 48 hours. Mouse IgG served as the negative control (NC) group. 'Input' refers to the DNA sample extracted from the total cell lysate. B-C, Expression of human *PGLYRP2* in liver tissues across different developmental stages (infant, fetal, adult) was assessed using RNA-seq data derived from human liver samples. D, The methylation status of the *PGLYRP2* promoter region was analyzed via bisulfite sequencing to ascertain epigenetic regulation patterns. E, Expression of *Dnmt3a* in mouse liver samples from various age groups was quantified by real-time PCR to evaluate age-related changes in methylation enzymes. F-G, The relationship between hepatic *PGLYRP2* expression and hepatic differentiation markers was examined. Analysis in F utilized data from TCGA, while in G, quantification was performed on samples from human normal tumor-adjacent liver tissues using quantitative RT-PCR (qRT-PCR). Data are represented as mean ± SD. One-way ANOVA with post hoc Bonferroni’s test (B and E), Student’s t-test (C) and Pearson's correlation coefficient (F and G) were used for statistical analysis. *$p < 0.05$; **$p < 0.001$; ns, not significant.

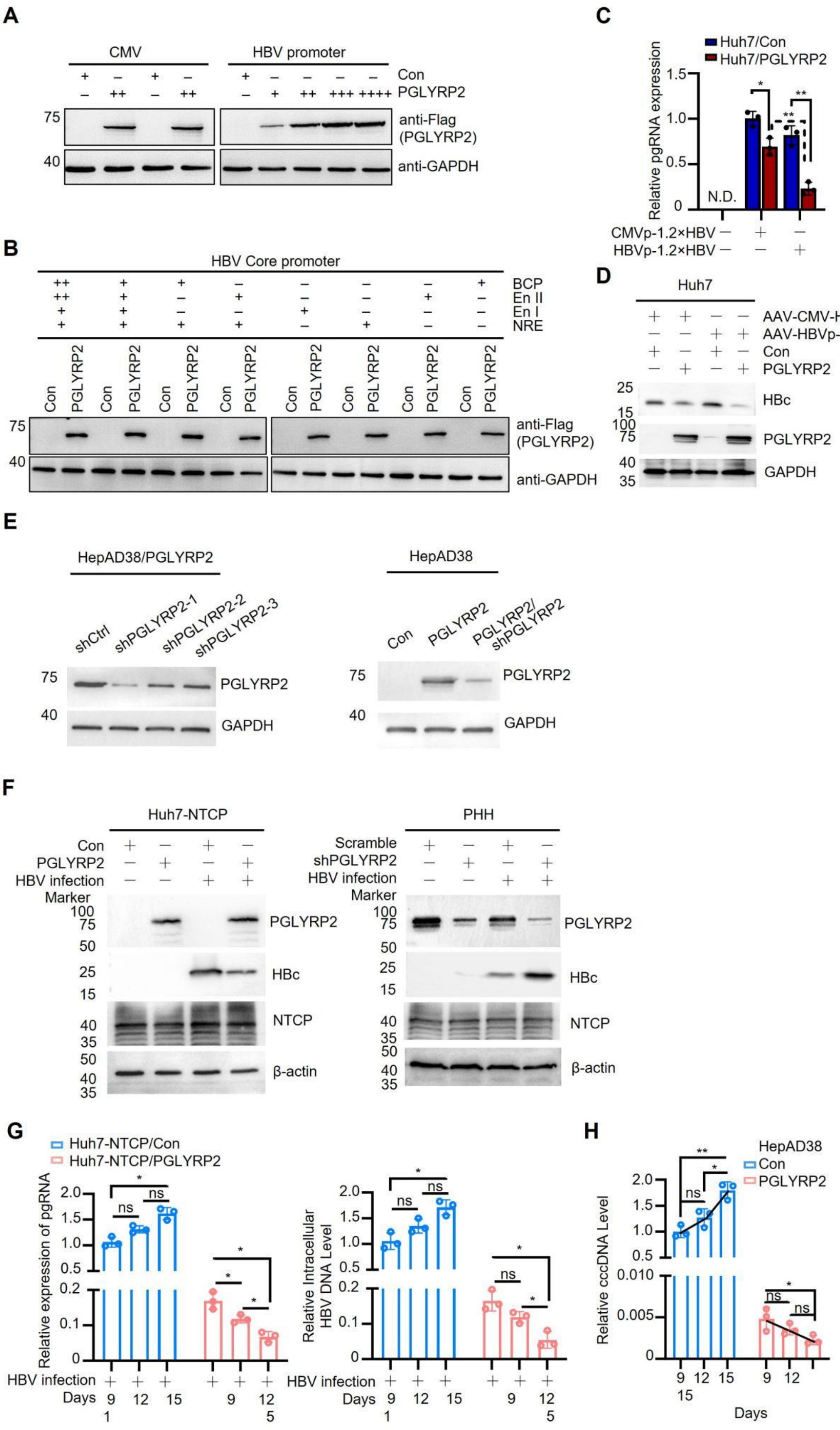
A
CMV
HBV promoter
Con
PGLYRP2
anti-Flag (PGLYRP2)
anti-GAPDH
C
Huh7/Con
Huh7/PGLYRP2
Relative pgRNA expression
N.D.
CMVp-1.2×HBV
HBVp-1.2×HBV
B
HBV Core promoter
BCP
En II
En I
NRE
D
Huh7
AAV-CMV-HBV1.2
AAV-HBVp-HBV1.2
HBc
GAPDH
E
HepAD38/PGLYRP2
HepAD38
shCtrl
shPGLYRP2-1
shPGLYRP2-2
shPGLYRP2-3
PGLYRP2/ shPGLYRP2
F
Huh7-NTCP
PHH
HBV infection
Marker
Scramble
shPGLYRP2
NTCP
β-actin
G
Huh7-NTCP/Con
Huh7-NTCP/PGLYRP2
Relative expression of pgRNA
Relative Intracellular HBV DNA Level
Days
H
HepAD38
Relative cccDNA Level
Days

**Fig. S2. PGLYRP2 restricts viral replication, related to Fig. 2.** A-B, PGLYRP2 expression levels in C3A/Con and C3A/PGLYRP2 cells were detected using Western blot. C-D, HBV plasmid (AAV-CMV promoter-1.2×HBV genome or AAV-HBV promoter-1.2×HBV genome) was transfected into stable cell line Huh7/Con or Huh7/PGLYRP2. After 6 days, pgRNA level and HBc expression was detected by real-time PCR (C) and western blot (D), respectively. E, Stable HepAD38/PGLYRP2 cells were transfected with a shPGLYRP2 1-3. At 72 h post-transfection, PGLYRP2 levels were detected by western blot (left). PGLYRP2 levels in stable HepAD38/Con, HepAD38/PGLYRP2 and HepAD38/PGLYRP2/shPGLYRP2 cells were detected by western blot (right). F, The expression of PGLYRP2, NTCP and HBc in HBV-infected stable Huh7-NTCP/Con or Huh7-NTCP/PGLYRP2 and PHH/Scramble or PHH/shPGLYRP2 cells was detected by western blot. G, Huh7-NTCP cells, either control or overexpressing PGLYRP2, were infected with HBV. Between nine and fifteen days post-infection, intracellular levels of pgRNA and HBV DNA were measured using real-time PCR. H, Quantitative Analysis of cccDNA. cccDNA from HepAD38 cells, treated with ExoI, ExoIII, and T5 nuclease, was quantitatively analyzed by PCR following 9-15 days of incubation in Tet-free medium. Bar values represent the mean of measurements from three biological replicates. Data are represented as mean ± SD. One-way ANOVA with post hoc Bonferroni's test (C) was used for statistical analysis. *$p < 0.05$; **$p < 0.001$; ns, not significant.

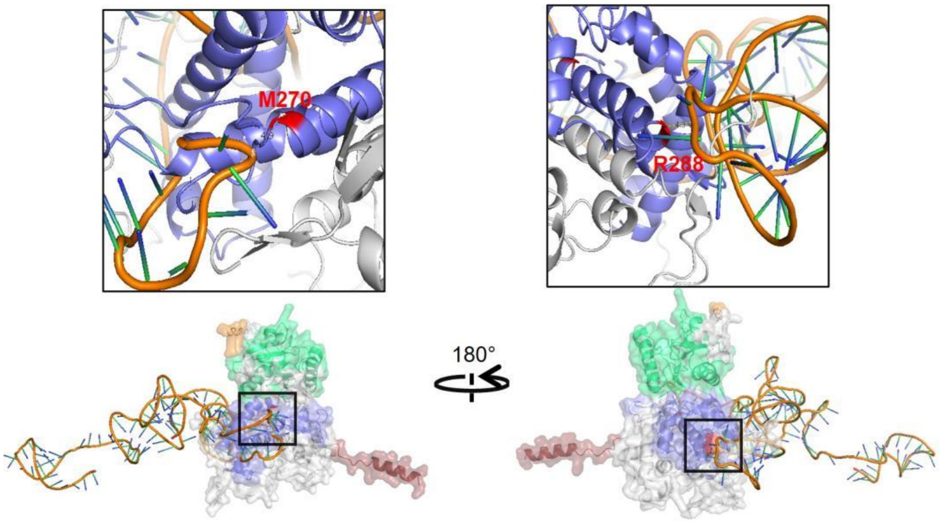


**Fig. S3. Structural and Protein-DNA Docking Analyses of PGLYRP2$^{209-377}$ and HBV DNA EnhII, related to Fig. 3.** Structural analyses using AlphaFold3 and Unafold predicted the conformations of PGLYRP2 and HBV DNA EnhII, respectively. Subsequent protein-DNA docking conducted with HDOCK identified potential interaction sites at residues M270 and R288.

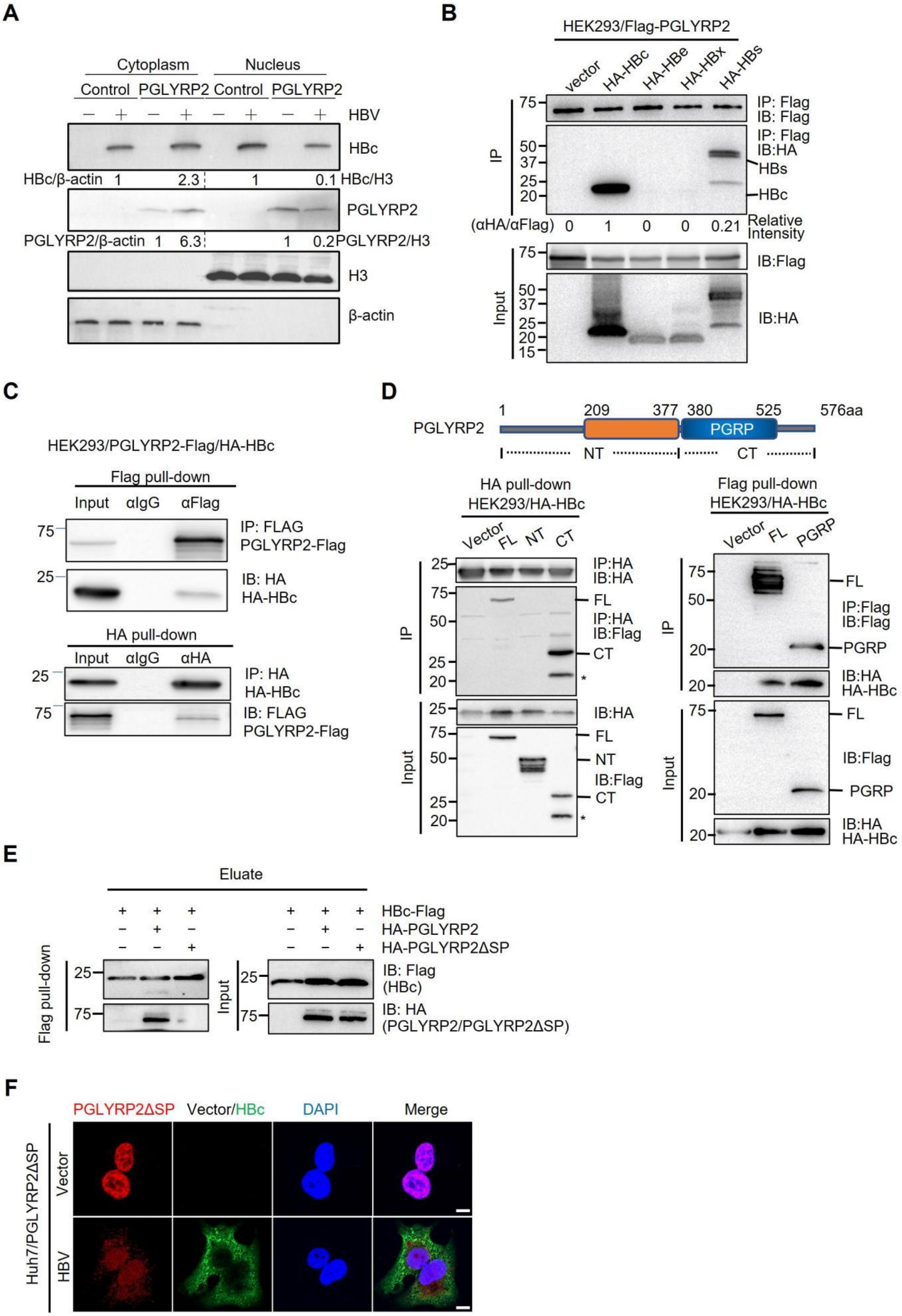

A
Cytoplasm
Nucleus
Control
PGLYRP2
Control
PGLYRP2
HBV
HBc
HBc/β-actin 1
2.3
1
0.1 HBc/H3
PGLYRP2
PGLYRP2/β-actin 1
6.3
1
0.2 PGLYRP2/H3
H3
β-actin
B
HEK293/Flag-PGLYRP2
vector
HA-HBc
HA-HBe
HA-HBx
HA-HBs
IP: Flag
IB: Flag
IP: Flag
IB:HA
HBs
HBc
IP
(αHA/αFlag) 0 1 0 0 0.21
Relative Intensity
IB:Flag
Input
IB:HA
C
HEK293/PGLYRP2-Flag/HA-HBc
Flag pull-down
Input
αIgG
αFlag
IP: FLAG
PGLYRP2-Flag
IB: HA
HA-HBc
HA pull-down
Input
αIgG
αHA
IP: HA
HA-HBc
IB: FLAG
PGLYRP2-Flag
D
PGLYRP2
1
209
377
380
525
576aa
PGRP
NT
CT
HA pull-down
HEK293/HA-HBc
Vector
FL
NT
CT
IP:HA
IB:HA
FL
IP:HA
IB:Flag
CT
IB:HA
NT
IB:Flag
Input
Flag pull-down
HEK293/HA-HBc
Vector
FL
PGRP
IP:Flag
IB:Flag
PGRP
IB:HA
HA-HBc
E
Eluate
HBc-Flag
HA-PGLYRP2
HA-PGLYRP2ΔSP
Flag pull-down
IB: Flag
(HBc)
IB: HA
(PGLYRP2/PGLYRP2ΔSP)
F
PGLYRP2ΔSP
Vector/HBc
DAPI
Merge
Huh7/PGLYRP2ΔSP
Vector
HBV

**Fig. S4. PGLYRP2 interacts with HBc through PGRP domain, related to Fig. 4.** A, Whole cell lysate prepared from 1.3×HBV genome construct-transfected Huh7/Con or Huh7/PGLYRP2 cells was separated into cytosolic and nuclear fractions. Histone H3 and β-actin were used as nucleus and cytoplasmic marker, respectively. B, PGLYRP2 expression construct was co-transfected with Con vector, HBc, HBe, HBx or HBs expression construct into HEK293 cells. After 48 h, cell lysates were harvested for co-IP using anti-FLAG antibody and blotted using the indicated antibodies. C, PGLYRP2 expression construct was co-transfected with HBc expression construct into HEK293 cells. After 48 h, cell lysates were harvested for co-IP using anti-FLAG antibody or anti-HA antibody and blotted using the indicated antibodies. D, Schematic representation of full-length PGLYRP2 (FL) and different truncations of PGLYRP2 (N-Terminal, NT; C-Terminal, CT; PGRP) (upper); HBc expression construct was co-transfected with Con vector, FL, NT, CT or PGRP domain of PGLYRP2 expression construct into HEK293 cells. After 48 h, cell lysates were harvested for co-IP using anti-HA (lower left) or anti-FLAG antibody (lower right) and blotted using the indicated antibodies. E, Western blot analyses of proteins eluted with 3×Flag peptides confirmed the formation of an extracellular complex between PGLYRP2 or PGLYRP2ΔSP and HBc. F, The localization of PGLYRP2ΔSP (Red) and HBc (Green) in HBV expression construct-transfected Huh7/PGLYRP2ΔSP cells was analyzed by immunofluorescence staining; Bar, 20 μm.

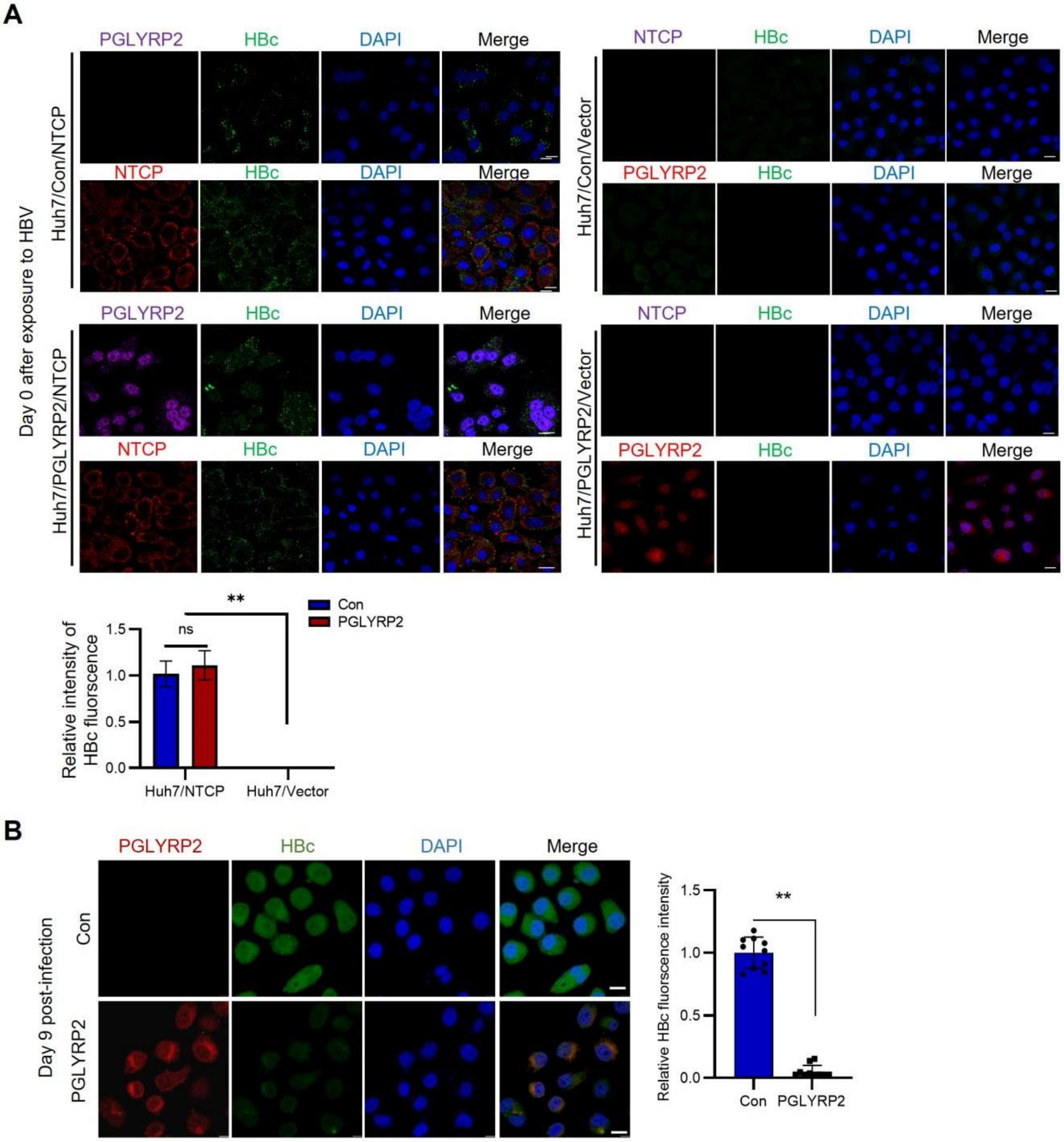


**Fig. S5. PGLYRP2 enhances HBV clearance, related to Fig. 5.** A, The expression of PGLYRP2, HBc and NTCP in Huh7/Con/NTCP, Huh7/PGLYRP2/NTCP cells (upper left), or Huh7/Con/vector, Huh7/PGLYRP2/vector cells (upper right) at day 0 after 48h exposure to HBV was analyzed by immunofluorescence staining. Quantification of HBc fluorescence intensity (lower left). B, The expression of PGLYRP2 (Red) and HBc (Green) in in Huh7/Con/NTCP or Huh7/PGLYRP2/NTCP cells at day 9-post HBV infection was analyzed by immunofluorescence staining (left). Quantification of HBc fluorescence intensity (right). Data are represented as mean ± SD. One-way ANOVA with post hoc Bonferroni's test (A) was used for statistical analysis. **$p$ < 0.001; ns, not significant.

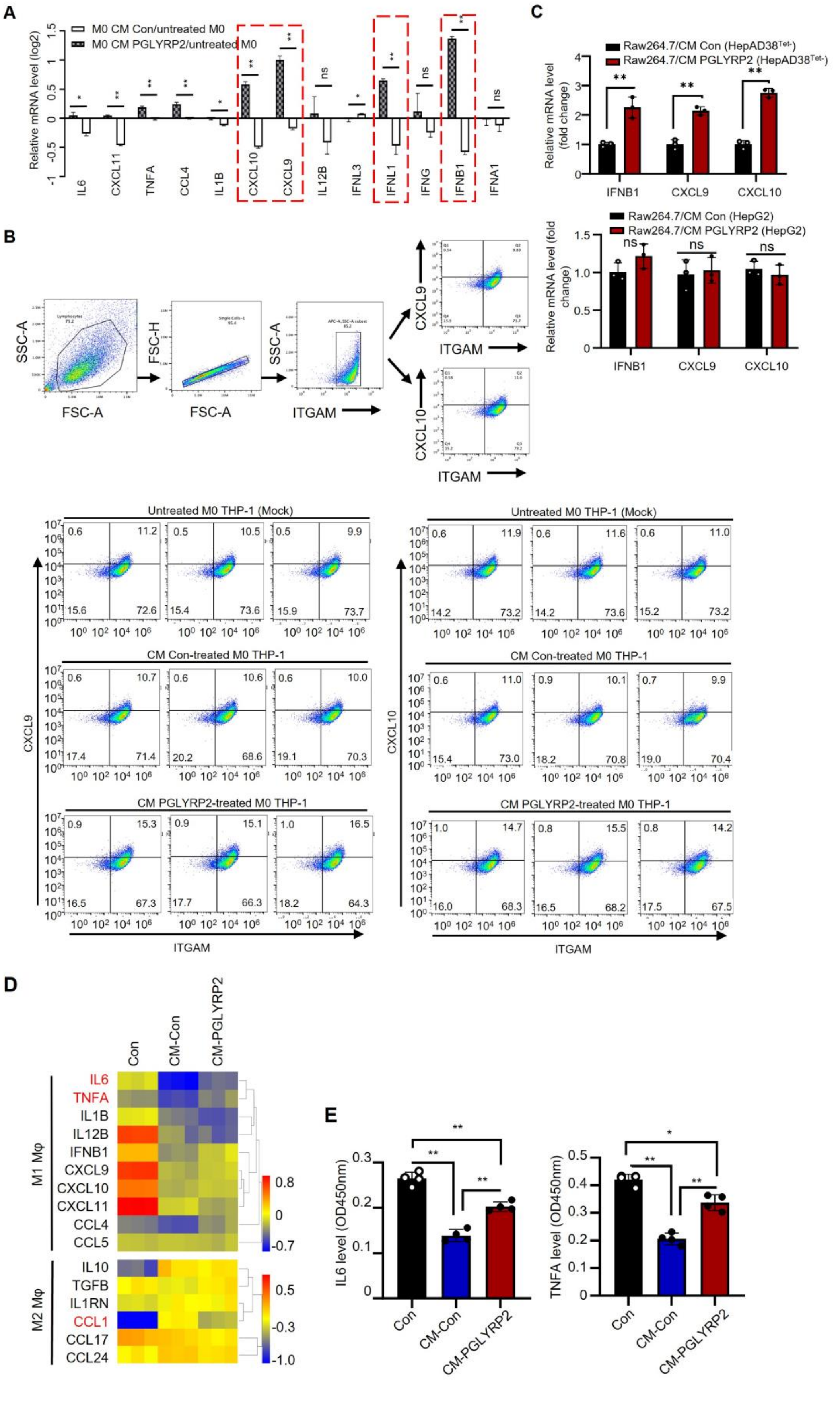
A
M0 CM Con/untreated M0
M0 CM PGLYRP2/untreated M0
Relative mRNA level (log2)
IL6
CXCL11
TNFA
CCL4
IL1B
CXCL10
CXCL9
IL12B
IFNL3
IFNL1
IFNG
IFNB1
IFNA1
ns
C
Raw264.7/CM Con (HepAD38Tet-)
Raw264.7/CM PGLYRP2 (HepAD38Tet-)
Relative mRNA level (fold change)
IFNB1
CXCL9
CXCL10
Raw264.7/CM Con (HepG2)
Raw264.7/CM PGLYRP2 (HepG2)
B
SSC-A
FSC-A
FSC-H
ITGAM
CXCL9
CXCL10
Untreated M0 THP-1 (Mock)
CM Con-treated M0 THP-1
CM PGLYRP2-treated M0 THP-1
D
Con
CM-Con
CM-PGLYRP2
M1 Mφ
M2 Mφ
IL10
TGFB
IL1RN
CCL1
CCL17
CCL24
CCL5
E
IL6 level (OD450nm)
TNFA level (OD450nm)

F

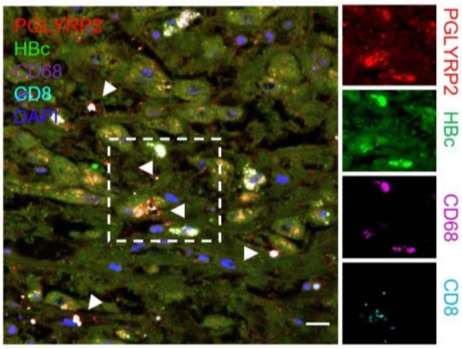
PGLYRP2
HBc
CD68
CD8
DAPI
PGLYRP2
HBc
CD68
CD8

G

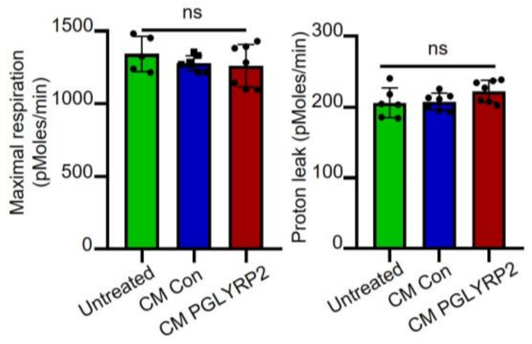
ns
Maximal respiration (pMoles/min)
1500
1000
500
0
Untreated
CM Con
CM PGLYRP2
ns
Proton leak (pMoles/min)
300
200
100
0
Untreated
CM Con
CM PGLYRP2

H

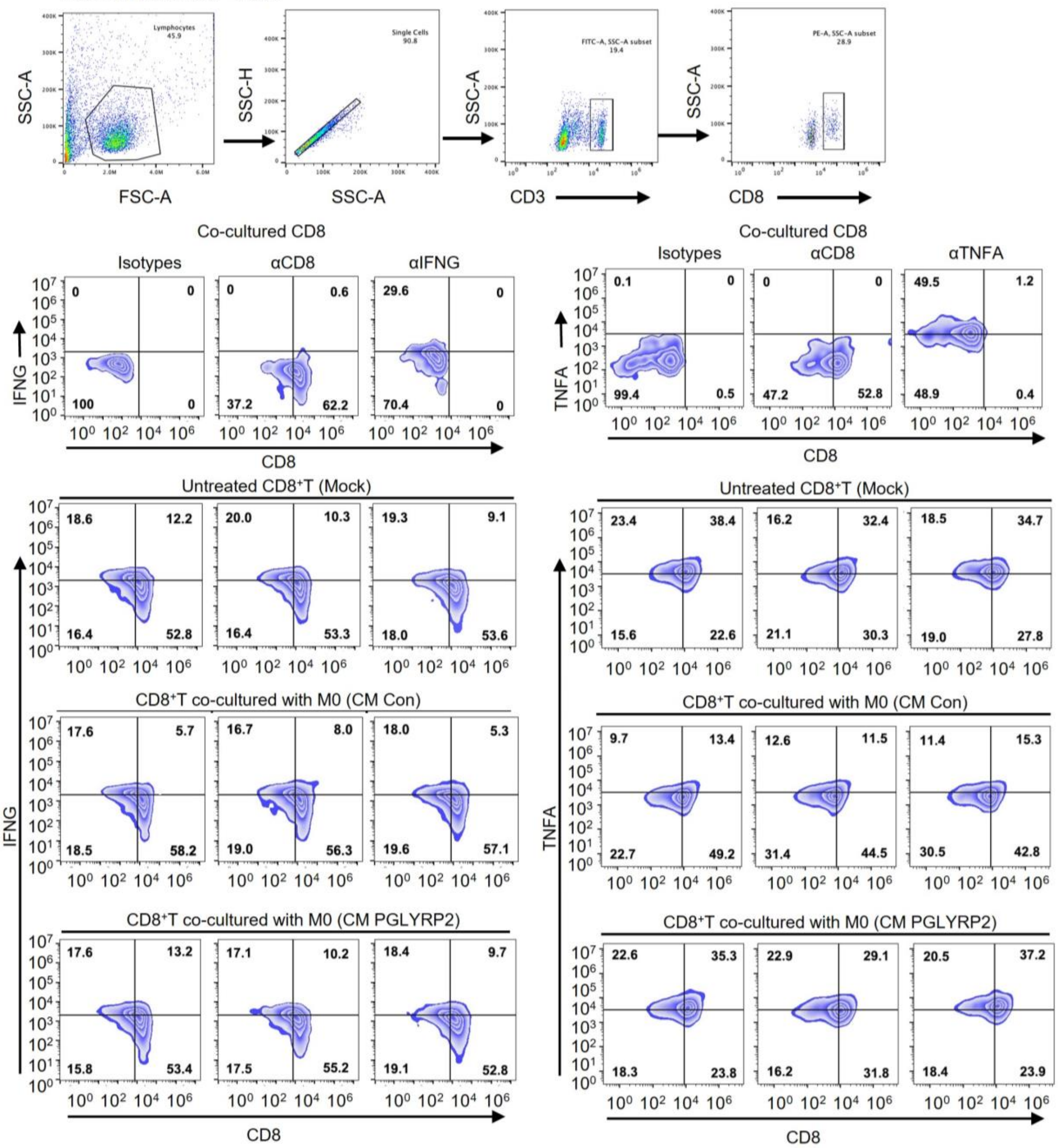
Human PBMC CD8⁺ T cells
Lymphocytes
45.9
Single Cells
90.8
FITC-A, SSC-A subset
19.4
PE-A, SSC-A subset
28.9
SSC-A
FSC-A
SSC-H
SSC-A
SSC-A
CD3
SSC-A
CD8
Co-cultured CD8
Isotypes
αCD8
αIFNG
IFNG
CD8
0
0
100
0
0
0.6
37.2
62.2
29.6
0
70.4
0
Co-cultured CD8
Isotypes
αCD8
αTNFA
TNFA
CD8
0.1
0
99.4
0.5
0
0
47.2
52.8
49.5
1.2
48.9
0.4
Untreated CD8⁺T (Mock)
18.6
12.2
16.4
52.8
20.0
10.3
16.4
53.3
19.3
9.1
18.0
53.6
CD8⁺T co-cultured with M0 (CM Con)
17.6
5.7
18.5
58.2
16.7
8.0
19.0
56.3
18.0
5.3
19.6
57.1
CD8⁺T co-cultured with M0 (CM PGLYRP2)
17.6
13.2
15.8
53.4
17.1
10.2
17.5
55.2
18.4
9.7
19.1
52.8
IFNG
CD8
Untreated CD8⁺T (Mock)
23.4
38.4
15.6
22.6
16.2
32.4
21.1
30.3
18.5
34.7
19.0
27.8
CD8⁺T co-cultured with M0 (CM Con)
9.7
13.4
22.7
49.2
12.6
11.5
31.4
44.5
11.4
15.3
30.5
42.8
CD8⁺T co-cultured with M0 (CM PGLYRP2)
22.6
35.3
18.3
23.8
22.9
29.1
16.2
31.8
20.5
37.2
18.4
23.9
TNFA
CD8

**Fig. S6. PGLYRP2 regulates the functional properties of immune effectors, related to Fig. 6.** A, the expression levels of specific cytokines or chemokines in THP-1 M0 macrophages treated with CM from HepAD38 Tet-off cells, either CM Con or CM PGLYRP2, were compared to those in untreated macrophages. B, Subtypes of THP-1 macrophages treated with CM from HepAD38 Tet-off cells, expressing $CXCL9^{+}ITGAM^{+}$ and $CXCL10^{+}ITGAM^{+}$, were analyzed using flow cytometry. C, The mRNA levels of IFNB1 and CXCL9/10 in HepAD38 $Tet^{-}$ derived CM (upper pannel) or HepG2-derived CM (lower pannel)-treated Raw264.7 were detected by real-time PCR assay. D, Hierarchical clustering illustrates the different expression of cytokines and chemokines in untreated Con, CM-Con or CM-PGLYRP2-treated THP-1 M1 or M2 macrophages. E, The levels of IL6 and TNFA in THP-1 M1 were detected by ELISA. F, Multiplex tissue IF staining for PGLYRP2, HBc, CD68 and CD8 in $HBV^{+}$ distal non-tumor liver tissues from HCC patients. G, The maximal respiration and proton leak were measured with the Seahorse MitoStress Test. H, Intracellular cytokine staining for IFNG and TNFA of the co-cultured $CD8^{+}$ T cells was analyzed by flow cytometry. Data are represented as mean ± SD. Student's t-test (A and B) and One-way ANOVA with post hoc Bonferroni's test (E) were used for statistical analysis. $^{**}p < 0.001$; ns, not significant.

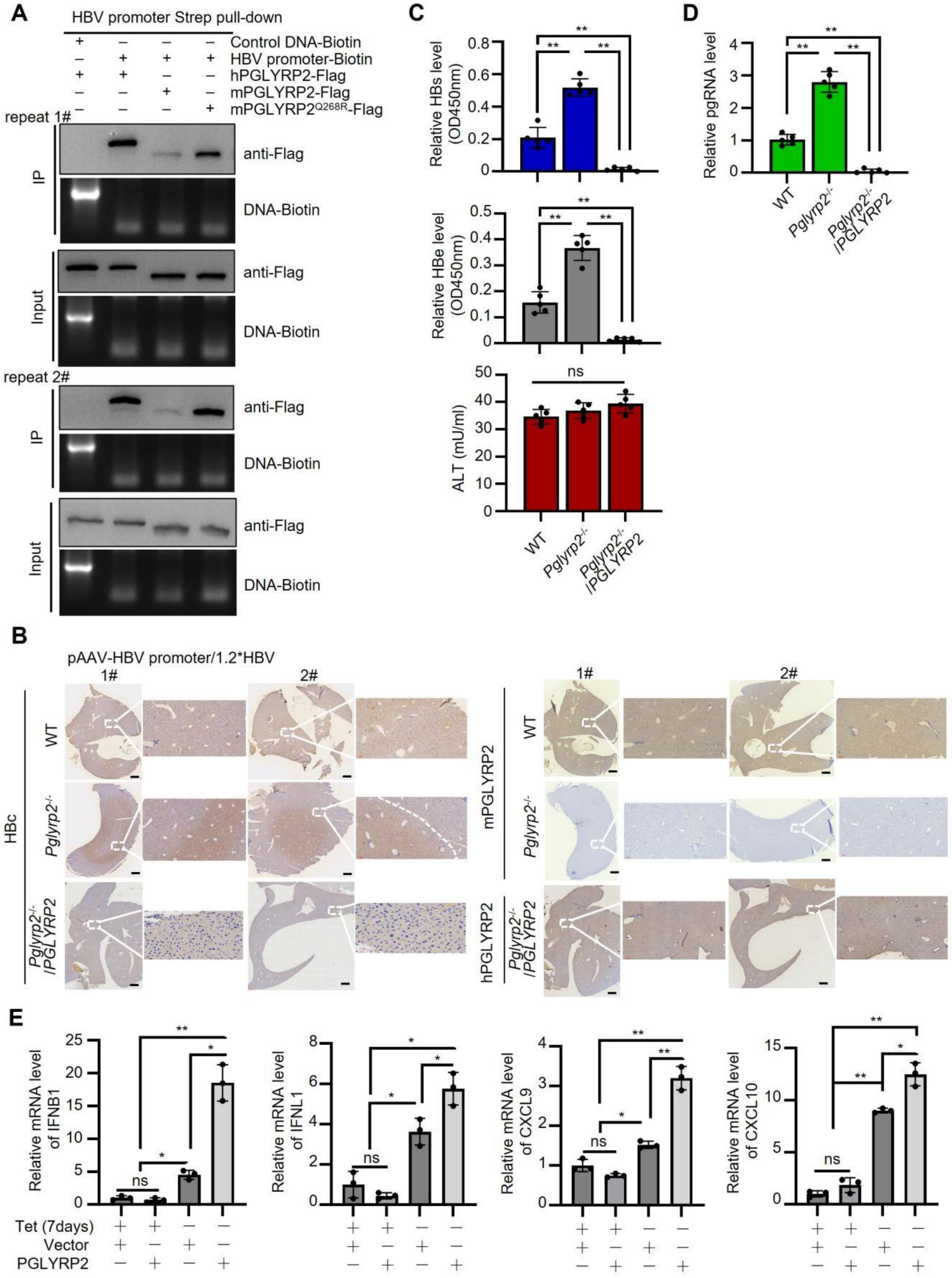
A
HBV promoter Strep pull-down
Control DNA-Biotin
HBV promoter-Biotin
hPGLYRP2-Flag
mPGLYRP2-Flag
mPGLYRP2Q268R-Flag
repeat 1#
repeat 2#
IP
Input
anti-Flag
DNA-Biotin
B
pAAV-HBV promoter/1.2*HBV
1#
2#
HBc
WT
Pglyrp2-/-
Pglyrp2-/- /PGLYRP2
mPGLYRP2
hPGLYRP2
C
Relative HBs level (OD450nm)
Relative HBe level (OD450nm)
ALT (mU/ml)
ns
D
Relative pgRNA level
E
Relative mRNA level of IFNB1
Relative mRNA level of IFNL1
Relative mRNA level of CXCL9
Relative mRNA level of CXCL10
Tet (7days)
Vector
PGLYRP2

**Fig. S7. HBV mouse model, related to Fig. 7.** A, Purification of HBV promoter-binding proteins (hPGLYRP2, mPGLYRP2, mPGLYRP2$^{Q268R}$) via DNA pull-down assays followed by Western blot and agarose gel analysis. Two additional replicates were conducted (related to Fig. 7C). B, IHC staining for HBc and PGLYRP2 in the liver tissues from mouse model with pAAV-HBV promoter/HBV1.2 at 6 weeks post-injection (Note: the IHC staining of HBc expression in mouse liver sections was also shown in Fig. 7F). C, the HBsAg, HBeAg and ALT levels in serum of the mouse model were detected by ELISA assay at 6 weeks post-injection. D, Intrahepatic pgRNA from mouse model were detected by real-time PCR. E, the expression of interferons (IFNB1, IFNL1) and interferon-stimulated genes (CXCL9, CXCL10) were analyzed using RT-PCR in HepaAD38 cells stably transfected with PGLYRP2 (HepaAD38/PGLYRP2) and control cells (HepaAD38/Con) after 7 days of culture in Tet-off medium. Data are represented as mean ± SD. One-way ANOVA with post hoc Bonferroni's test (B and C) were used for statistical analysis. *$p < 0.05$; **$p < 0.001$; ns, not significant.

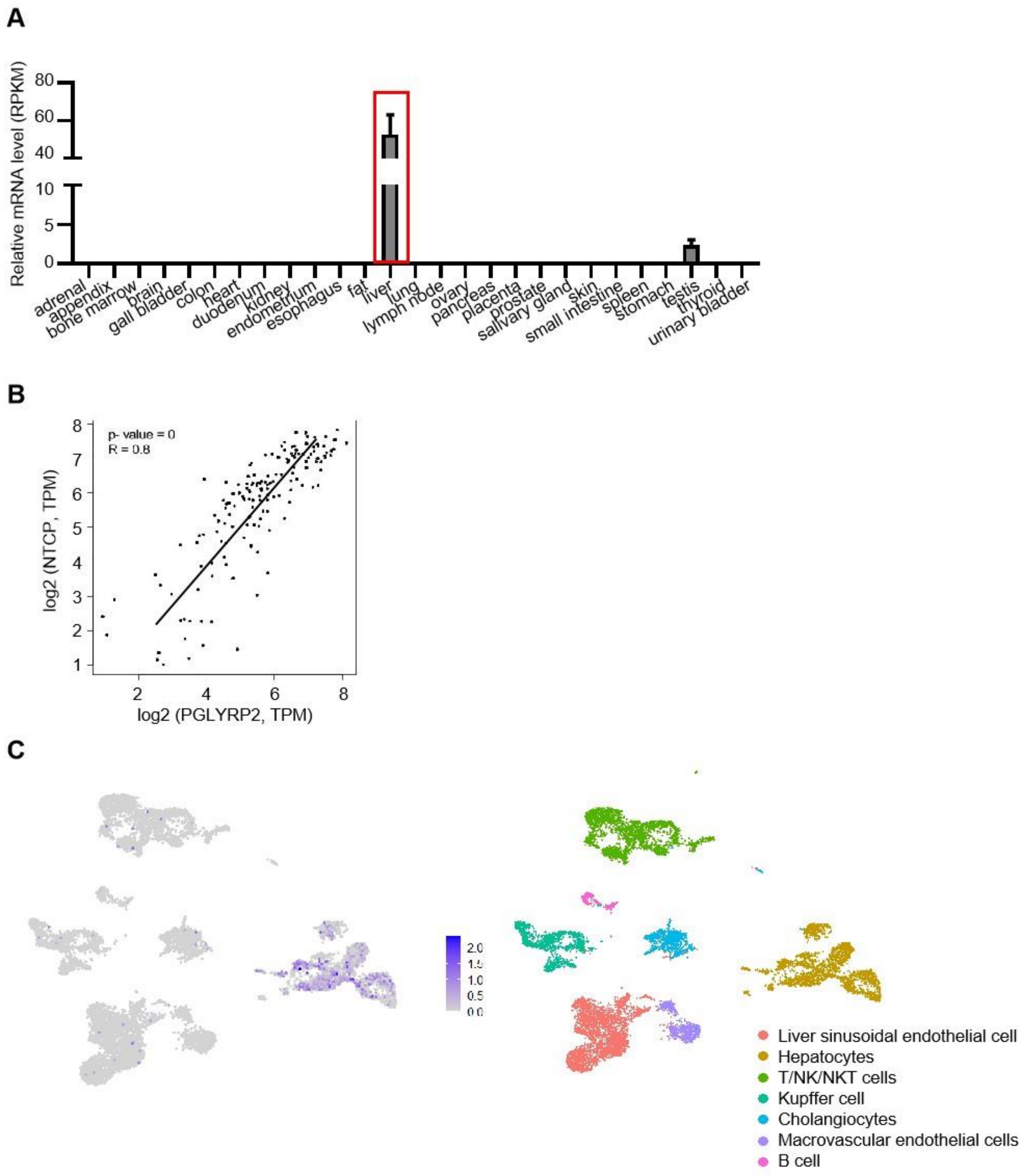


**Fig. S8. Heterogeneity of PGLYRP2 expression.** A-B, PGLYRP2 is predominantly synthesized in human liver tissues (A) and its expression is positively correlated with NTCP (B) revealed by RNA-seq data. C, Single-cell RNA-seq data showed that individual hepatocyte expresses distinct level of *PGLYRP2*. Pearson's correlation coefficient (B) were used for statistical analysis.

**Table S1. The identified_data by LC-MS/MS Analysis**

| Gene names | MW [kDa] | Protein score | Sequence coverage (%) | # Unique Peptides | # Peptides | # PSMs |
|---|---|---|---|---|---|---|
| HRNR | 282.23 | 930.45 | 16.25 | 15 | 15 | 25 |
| ACACB | 276.37 | 1460.99 | 24.65 | 44 | 53 | 59 |
| ACACA | 265.39 | 843.78 | 16.50 | 25 | 34 | 36 |
| MYH9 | 226.39 | 941.03 | 15.61 | 23 | 23 | 31 |
| CPS1 | 164.84 | 254.66 | 7.07 | 9 | 9 | 9 |
| PC | 129.55 | 2159.68 | 45.93 | 41 | 41 | 74 |
| DDB1 | 126.89 | 187.92 | 9.65 | 9 | 9 | 9 |
| PARP1 | 113.01 | 598.89 | 21.10 | 18 | 18 | 22 |
| PCCA | 80.01 | 1256.39 | 43.82 | 27 | 27 | 37 |
| PGLYRP2 | 62.22 | 886.82 | 50.35 | 30 | 30 | 56 |
| MCCC2 | 61.29 | 923.45 | 35.88 | 19 | 19 | 29 |
| PCCB | 58.18 | 1021.14 | 50.65 | 19 | 19 | 27 |
| NFIC | 55.64 | 176.74 | 11.42 | 4 | 5 | 7 |
| VIM | 53.62 | 176.88 | 16.52 | 8 | 8 | 9 |
| HNRNPK | 50.94 | 271.07 | 20.95 | 9 | 9 | 11 |
| CMAS | 48.35 | 136.91 | 11.98 | 5 | 5 | 5 |
| DDB2 | 47.83 | 57.61 | 6.09 | 2 | 2 | 2 |
| HNRNPDL | 46.41 | 113.16 | 12.86 | 3 | 5 | 6 |
| HNRNPA3 | 39.57 | 249.95 | 22.49 | 6 | 7 | 9 |
| YBX1 | 35.90 | 886.82 | 58.33 | 12 | 12 | 17 |
| RPL8 | 28.01 | 33.06 | 6.23 | 1 | 1 | 1 |
| HMGB1 | 24.88 | 350.91 | 25.58 | 7 | 7 | 10 |
| RPS8 | 24.19 | 54.97 | 5.29 | 1 | 1 | 1 |
| HMGB2 | 24.02 | 91.31 | 11.96 | 3 | 3 | 3 |

| | | | | | | |
|---|---|---|---|---|---|---|
| RPL19 | 23.45 | 72.50 | 8.67 | 1 | 1 | 1 |
| MGMT | 21.63 | 42.77 | 6.28 | 1 | 1 | 1 |
| RPL17 | 21.38 | 49.56 | 5.43 | 1 | 1 | 1 |
| SRSF3 | 19.32 | 58.96 | 14.02 | 2 | 2 | 2 |
| CIRBP | 18.64 | 97.34 | 14.53 | 2 | 2 | 2 |
| RPS27A | 17.95 | 71.75 | 16.03 | 2 | 2 | 2 |
| RPL24 | 17.77 | 41.21 | 5.73 | 1 | 1 | 1 |
| RPL29 | 17.74 | 60.92 | 9.43 | 1 | 1 | 1 |
| SUB1 | 14.39 | 480.98 | 44.88 | 6 | 6 | 18 |
| HMGA1 | 11.67 | 27.15 | 8.41 | 1 | 1 | 1 |

**Table S2. Analysis of *PGLYRP2* expression and hepatic differentiation markers**

| **Hepatic differentiation maker** | ***p*-Value** | **R** |
|---|---|---|
| TTR | $p<0.001$ | R=0.94 |
| HNF4A | $p<0.001$ | R=0.88 |
| ALB | $p<0.001$ | R=0.86 |
| DPP4 | $p<0.001$ | R=0.86 |
| APOF | $p<0.001$ | R=0.86 |
| AFP | $p=0.002$ | R=0.77 |
| CPS1 | $p=0.002$ | R=0.63 |

**Table S3. IHC staining for PGLYRP2 and HBc in paraffin-embedded human liver tissues**

| Case No. | PGLYRP2 staining | PGLYRP2 intensity | HBc staining | HBc intensity |
|---|---|---|---|---|
| 6289D | 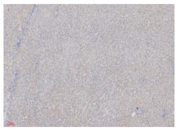 | + +/- | 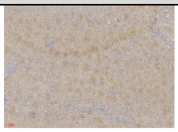 | +++ +/- |
| 7214F | 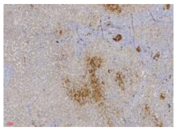 | +++ | 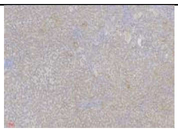 | + |
| 7855A | 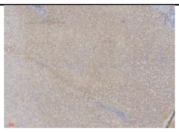 | ++ +/- | 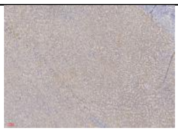 | ++ |
| 9966A | 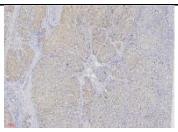 | + +/- | 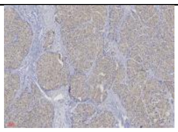 | ++ |
| 4805B | 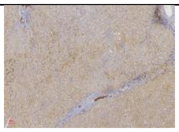 | +++ | 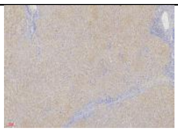 | + |
| 6775D | 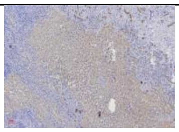 | + +/- | 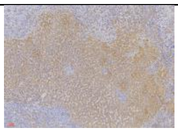 | ++ +/- |
| 4840E | 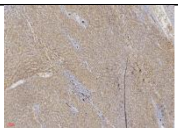 | +++ | 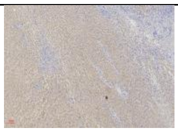 | + |
| 1592F | 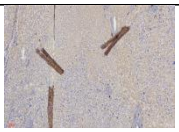 | ++ | 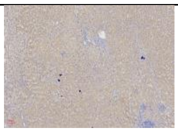 | + |
| 4619C | 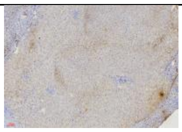 | + | 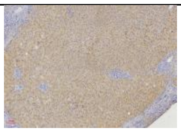 | +++ |
| 1037O | 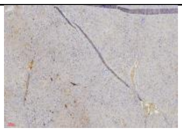 | + | 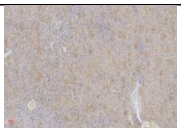 | ++ +/- |

| | | | | |
|---|---|---|---|---|
| 8020D | 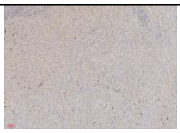 | + +/- | 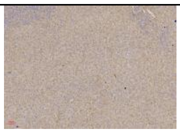 | +++ +/- |
| 4538F | 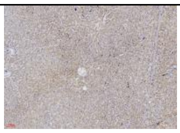 | ++ | 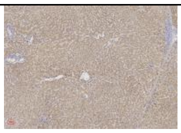 | +++ |
| 8709E | 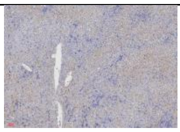 | + | 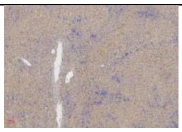 | ++++ |
| 882303 | 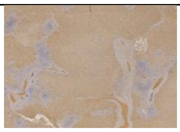 | ++++ | 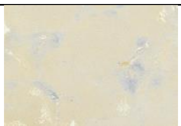 | +/- |
| 879115 | 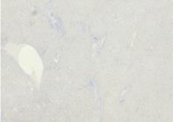 | +/- | 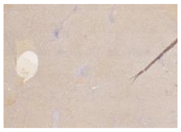 | ++ |
| 4594D | 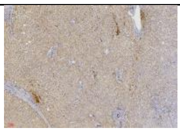 | ++ | 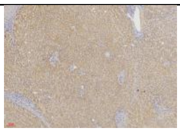 | +++ |
| 0789B | 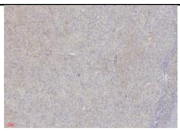 | + +/- | 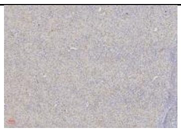 | + +/- |

Note: Cases #4840E and #4619C are also shown in Fig. 5B.

**Table S4. PHH donor information**

| Case No. | Age | Gender | Hepatitis B | Reason for liver resection |
|---|---|---|---|---|
| 8103C | 42 | Male | - | Hepatocellular carcinoma |
| 3542F | 58 | Female | - | Hepatocellular carcinoma |

**Table S5. Reagents**

| REAGENT or RESOURCE | SOURCE | IDENTIFIER |
|---|---|---|
| Antibodies | | |
| CD3-APC | BioLegend | Cat#317318 |
| CD8-FITC | BioLegend | Cat#301006 |
| ITGAM-FITC | BioLegend | Cat#301329 |
| CD68-PE | BioLegend | Cat#333807 |
| CXCL9-APC | BioLegend | Cat#357906 |
| CXCL10-APC | BioLegend | Cat#519506 |
| IFNG-APC | BioLegend | Cat#562017 |
| IFNG-PE | BioLegend | Cat#502509 |
| TNFA-APC | BioLegend | Cat#502912 |
| HBC antibody | biorbyt | Cat#orb99015 |
| HA-Tag Mouse mAb | Cell Signaling | Cat#2367 |
| GAPDH XP Rabbit mAb | Cell Signaling | Cat#5174 |
| DYKDDDDK Tag Rabbit mAb | Cell Signaling | Cat#14793 |
| HA-Tag Rabbit mAb | Cell Signaling | Cat#3724 |
| Alexa Fluor® 594 goat anti-mouse IgG (H+L) | Thermo Fisher Scientific | Cat#A11005 |
| Alexa Fluor® 488 goat anti-mouse IgG (H+L) | Thermo Fisher Scientific | Cat#A11001 |
| goat anti-rabbit IgG-HRP | absin | Cat#abs20040 |
| goat anti-mouse IgG-HRP | absin | Cat#abs2039ss |
| HBc monoclonal antibody | biorbyt | Cat#orb99015 |
| CD68 monoclonal antibody | abcam | Cat#ab955 |
| CD8A polyclonal antibody | ABclonal | Cat#A11856 |

| | | |
|---|---|---|
| NTCP polyclonal antibody | abcam | Cat#ab131084 |
| hPGLYRP2 polyclonal antibody | Novus | Cat#NBP2-32042 |
| mPGLYRP2 monoclonal antibody | R&D Systems | Cat#MAB4704 |
| secondary antibodies (goat anti-rat conjugated with HRP） | ZSGB-BIO | Cat#PV-9004 |
| secondary antibodies (goat anti-mouse conjugated with HRP） | ZSGB-BIO | Cat#PV-6002 |
| secondary antibodies (goat anti-rabbit conjugated with HRP） | ZSGB-BIO | Cat#PV-6001 |
| Bacterial and virus strains | | |
| Hepatitis B virus | HepAD38 | N/A |
| pAAV2/8-hPGLYRP2 Particle | This paper | N/A |
| Human PGLYRP2 shRNA Lentiviral Particle | OriGene Technologies | Cat#TL302528V |
| Biological samples | | |
| Human liver tissue specimens from distal non-tumor liver tissues of liver cancer patients | Harbin Medical University Cancer Hospital | http://hmucancerhospital.org.cn/ |
| Primary human hepatocytes (PHHs) | Harbin Medical University Cancer Hospital | http://hmucancerhospital.org.cn/ |

| | | |
|---|---|---|
| Chemicals, peptides, and recombinant proteins | | |
| human IFNG (CHO-expressed) | MCE | Cat# HY-P7025A |
| human IL4 (CHO-expressed) | MCE | Cat# HY-P7219 |
| 3*Flag Peptide | Beyotime | Cat# P9801 |

| | | |
|---|---|---|
| RPMI 1640 | gibco | Cat# 21870-076 |
| RPMI Medium Modified | Cytiva | Cat# SH30809.01 |
| PBS | Cytiva | Cat# SH30256.01 |
| DME/F-12 1:1 | Cytiva | Cat# SH30023.01 |
| DMEM/HIGH GLUCOSE | Cytiva | Cat# SH30022.01 |
| Fetal bovine serum (FBS) | ScienceCell | Cat# 0500 |
| Fetal bovine serum (FBS) | BI | Cat. #04-001-IACS-500ml |
| Anti-Anti (100×) | Gibco | Cat# 15240-062 |
| GlutaMAX | Gibco | Cat#35050061 |
| Paraformaldehyde | Sandon Biotech | Cat# 30525-89-4 |
| Ex-MEM | SEVEN BioTECH | Cat# SC105-01 |
| Puromycin Dihydrochloride | biosharp | Cat# BS111-25mg |
| Triton-X 100 | biosharp | Cat# 9002-93-1 |
| LPS | Sigma | Cat# L6529-1MG |
| DAPI | MCE | Cat# 2387906-44-5 |
| G418 | Biosharp | Cat# BS150-5g |
| Trizol | ThermoFisher | Cat# 15596026 |
| Lipofectamine 3000 transfection reagent | Invitrogen | Cat# L3000015 |
| Quick Ligase | NEB | Cat# M2200L |
| UltraSYBR Mixture (Low ROX) | CWBIO | Cat#CW2601M |
| Pierce Universal Nuclease | ThermoFisher | Cat# 88702 |
| Protease inhibitor | Cocktail Biosharp | Cat# BL630B |
| 2×GoldStar Best MasterMix(Dye) | CWBIO | Cat# CW0655M |
| DMSO | SIGMA | Cat# D8418-100ml |
| Streptavidin Magnetic Beads | MCE | Cat# HY-K0208 |

| | | |
|---|---|---|
| Anti-HA Magnetic Beads | Thermo | Cat# 88836 |
| Anti-DYKDDDDK Magnetic Agarose | Thermo | Cat# A36797 |
| Critical commercial assays | | |
| Seahorse XF RPMI | Agilent Technologies | Cat#103681-100 |
| Multiplex immunofluorescence kit (Novo Light TSA，human) | WiSee Biotechnology | Cat# H-D110051 |
| Deposited data | | |
| N/A | | |
| Experimental models: Cell lines | | |
| HEK293T | ATCC | Cat#CRL-3216 |
| THP-1 | ATCC | Cat#TIB-202 |
| C3A | ATCC | Cat#HB-8065 |
| Huh7 | cell bank of type culture collection of the Chinese Academy of Sciences (Shanghai, China) | Cat#SCSP-526 |
| Experimental models: Organisms/strains | | |
| Mouse: C57BL/6J Wild type | Cyagen Biosciences | Strain: C57BL/6J |
| Mouse: C57BL/6J *pglyrp2*$^{-/-}$ | Cyagen Biosciences | Strain: C57BL/6J |

| Oligonucleotides | | |
|---|---|---|
| cccDNA-specific primer_F | Ruibiotech | GTCTGTGCCTTCTCAT CTGC |
| cccDNA-specific primer_R | Ruibiotech | ACAAGAGATGATTAG GCAGAGG |
| HBV-rcDNA_RT_F | Ruibiotech | GGAGGCTGTAGGCAT AAATTGG |
| HBV-rcDNA_RT_R | Ruibiotech | CACAGCTTGGAGGCT TGAAC |
| HBV-PreC/C_RT_F | Ruibiotech | AGACCACCAAATGCC CCTATC |
| HBV-PreC/C_RT_R | Ruibiotech | TCTGCGAGGCGAGGG AGTTC |
| HBV-S_RT_F | Ruibiotech | TGGCCAAAATTCGCA GTCCC |
| HBV-S_RT_R | Ruibiotech | GAAGAACCAACAAGA AGATGAGGC |
| HBV-X_RT_F | Ruibiotech | CATGGAACCACCGTG AACG |
| HBV-X_RT_R | Ruibiotech | CCAATTTATGCCTACA GCCTCCT |
| HBV-pgRNA _RT_F | Ruibiotech | TGTTCAAGCCTCCAAG CT |
| HBV-pgRNA _RT_R | Ruibiotech | GGAAAGAAGTCAGAA GGCAA |
| β-actin-F | Ruibiotech | CATGTACGTTGCTATC CAGGC |

| | | |
|---|---|---|
| β-actin-R | Ruibiotech | CTCCTTAATGTCACGC ACGAT |
| hIL6-RT-F | Ruibiotech | ACTCACCTCTTCAGAA CGAATTG |
| hIL6-RT-R | Ruibiotech | CCATCTTTGGAAGGTT CAGGTTG |
| hCXCL11-RT-F | Ruibiotech | GACGCTGTCTTTGCAT AGGC |
| hCXCL11-RT-R | Ruibiotech | GGATTTAGGCATCGTT GTCCTTT |
| hTNFA-RT-F | Ruibiotech | GAGGCCAAGCCCTGG TATG |
| hTNFA-RT-R | Ruibiotech | CGGGCCGATTGATCTC AGC |
| hCCL4-RT-F | Ruibiotech | CTGTGCTGATCCCAGT GAATC |
| hCCL4-RT-R | Ruibiotech | TCAGTTCAGTTCCAGG TCATACA |
| hCCL1-RT-F | Ruibiotech | CTCATTTGCGGAGCA AGAGAT |
| hCCL1-RT-F | Ruibiotech | GCCTCTGAACCCATCC AACTG |
| hCCL1-RT-R | Ruibiotech | ACATCATCCCTACGG GCTCT |
| hCCL24-RT-F | Ruibiotech | CTTGGGGTCGCCACA GAAC |

| | | |
|---|---|---|
| hCCL24-RT-R | Ruibiotech | TAAGAGGGCCAAAGA TGCCTT |
| hIFNL3-RT-F | Ruibiotech | CTGGTCCAAGACATC CCCC |
| hIFNL3-RT-R | Ruibiotech | GCTTGGATTCCTACAA AGAAGCA |
| hIFNB1-RT-F | Ruibiotech | ATAGATGGTCAATGC GGCGTC |
| hIFNB1-RT-R | Ruibiotech | CCAGTAGTGAGAAAG GGTCGC |
| hCXCL9-RT-F | Ruibiotech | AGGGCTTGGGGCAAA TTGTT |
| hCXCL9-RT-R | Ruibiotech | GTGGCATTCAAGGAG TACCTC |
| hCXCL9-RT-F | Ruibiotech | TGATGGCCTTCGATTC TGGATT |
| hCXCL9-RT-R | Ruibiotech | CACATTGGCAGGTTC AAATCTCT |
| hCXCL10-RT-F | Ruibiotech | CCAGCGGACTCCTTTT TGG |
| hCXCL10-RT-R | Ruibiotech | CTAATGGTGGAAACC CACAACG |
| hIFNL1-RT-F | Ruibiotech | TATCGCCAGGAATTGT TGCTG |
| hIFNL1-RT-R | Ruibiotech | CCAGCAGTCGTCTTTG TCAC |

| | | |
|---|---|---|
| hTGF-Beta-RT-F | Ruibiotech | CTCTGGGTTGGCACACACTT |
| hTGF-Beta-RT-R | Ruibiotech | ATGATGGCTTATTACAGTGGCAA |
| hCCL5-RT-F | Ruibiotech | GTCGGAGATTCGTAGCTGGA |
| hCCL5-RT-R | Ruibiotech | TCAAGGCGCATGTGAACTCC |
| hIL1B-RT-F | Ruibiotech | GATGTCAAACTCACTCATGGCT |
| hIL1B-RT-R | Ruibiotech | TGCCCATTGAGGTCATGGTG |
| hIL10-RT-F | Ruibiotech | CTTGGGTGGGTCAGGTTTGA |
| hIL10-RT-R | Ruibiotech | TCGGTAACTGACTTGAATGTCCA |
| hIL12B-RT-F | Ruibiotech | TCGCTTCCCTGTTTTAGCTGC |
| hIL12B-RT-R | Ruibiotech | CATTGAGCCTCATGCTCTGTT |
| hIFNG-RT-F | Ruibiotech | CGCTGTCTGAGCGGATGAA |
| hIFNG-RT-R | Ruibiotech | GCCTCGCCCTTTGCTTTACT |
| hIL1Rantagonist-RT-F | Ruibiotech | CTGTGGGTCTCAGGGAGATCA |

| | | |
|---|---|---|
| hIL1Rantagonist-RT-R | Ruibiotech | TGCTGCCACTAATGCTGATGT |
| hIFNA1-RT-F | Ruibiotech | CTCAGGAACCAATCTTTGCACT |
| hIFNA1-RT-R | Ruibiotech | ACTGAAACGGAATGCCTTCCT |
| hCXCL17-RT-F | Ruibiotech | CCTCACTCGTACAGTGCCA |
| hCXCL17-RT-R | Ruibiotech | GGCCCGAGTACAAGAACCG |
| hCD40-RT-F | Ruibiotech | TCGTATGTGCCCTCGTCAGAT |
| hCD40-RT-R | Ruibiotech | TCAAGCAGTATTGGAACAGAGGA |
| hCD80-RT-F | Ruibiotech | CAGGAGGCTGCGGACTTTTT |
| hCD80-RT-R | Ruibiotech | TGGTTGTTGAAAGTCAATGGCT |
| hCD209-RT-F | Ruibiotech | CTCAGATGCCTTCACCTTGTTT |
| hCD209-RT-R | Ruibiotech | TGGGTGGAATGAGACTATTGTTG |
| hCD200-RT-F | Ruibiotech | CTCCCACGTCAATCTTTCCTC |
| hCD200-RT-R | Ruibiotech | GGAGTTCGAGGAACCCTAGTG |

| | | |
|---|---|---|
| mIFNB1-RT-F | Ruibiotech | GGGATTTGTAGTGGA TCGTGC |
| mIFNB1-RT-R | Ruibiotech | CCAAGTGCTGCCGTC ATTTTC |
| mCXCL9-RT-F | Ruibiotech | GGCTCGCAGGGATGA TTTCAA |
| mCXCL9-RT-R | Ruibiotech | TGACCTCAACTACATG GTCTACA |
| mCXCL10-RT-F | Ruibiotech | CTTCCCATTCTCGGCC TTG |
| mCXCL10-RT-R | Ruibiotech | CATGTACGTTGCTATC CAGGC |
| mGAPDH-RT-F | Ruibiotech | CTCCTTAATGTCACGC ACGAT |
| Recombinant DNA | | |
| pLV-3Flag-hPGLYRP2 | This paper | N/A |
| pLV-3Flag-hPGLYRP2$^{209-377}$ | This paper | N/A |
| pLV-3Flag-mPGLYRP2 | This paper | N/A |
| pLV-3Flag-mPGLYRP2$^{Q268R}$ | This paper | N/A |
| pCMV-HA-HBc | This paper | N/A |
| pCMV-HA-HBc C61G | This paper | N/A |
| pCMV-HA-HBc Y132A | This paper | N/A |
| Software and algorithms | | |
| FlowJo | BD Biosciences | Version 10.8.1 |
| ZEN (blue edition) | Zeiss ZEN | Version 2.0 |

| | | |
|---|---|---|
| GraphPad Prism | GraphPad Software Inc. | Version 9.2 |
| Other | | |
