# Supplementary material for: PGLYRP2 drives hepatocyte-intrinsic innate immunity by trapping and clearing hepatitis B virus
Source: J Clin Invest. 2025 Feb 13;135(8):e188083. doi: 10.1172/JCI188083 (PMC11996887; doi:10.1172/JCI188083)
Supplement: Unedited blot and gel images [file jci-135-188083-s223.pdf]

A

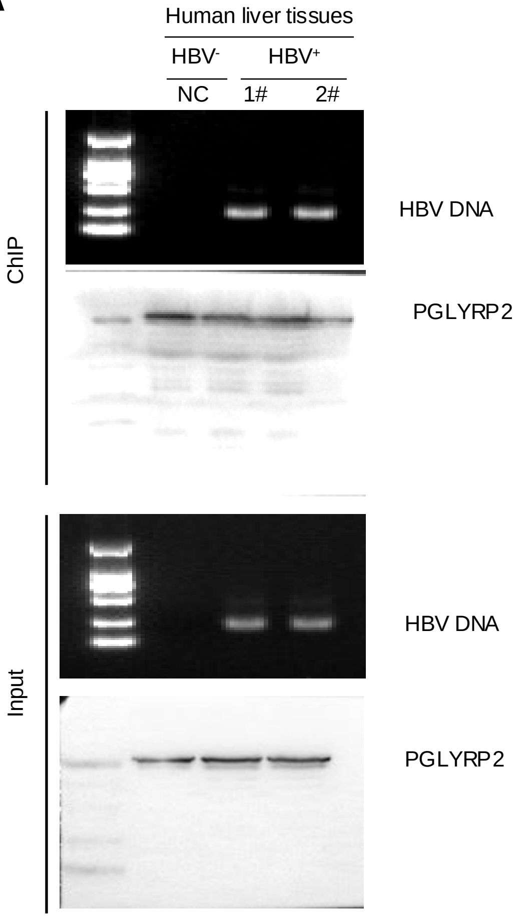

Full unedited blot/gel for Figure 1C

B

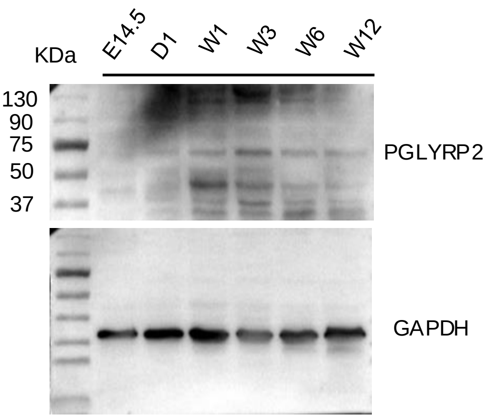

Full unedited blot for Figure 1F

C

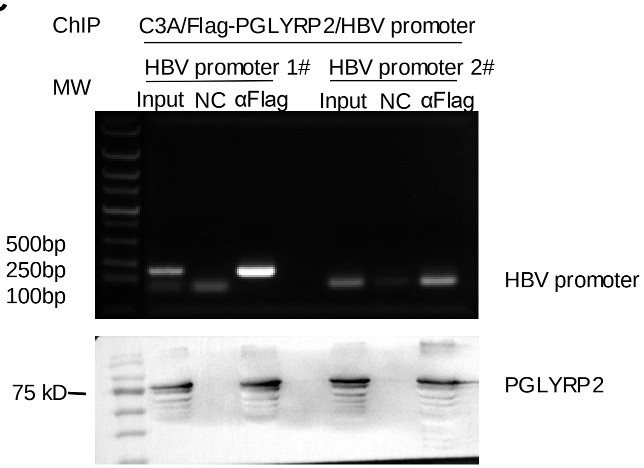

Full unedited blot/gel for Figure S1A

Full, unedited figures related to Figure 1 and Figure S1

A

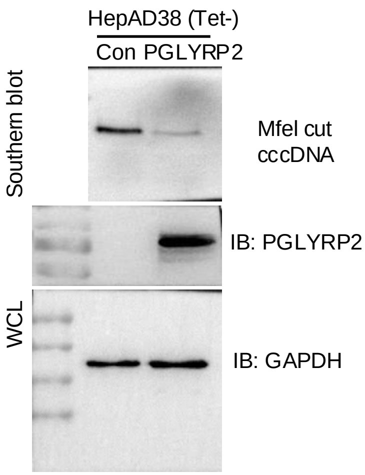

Full unedited blot for Figure 2K

B

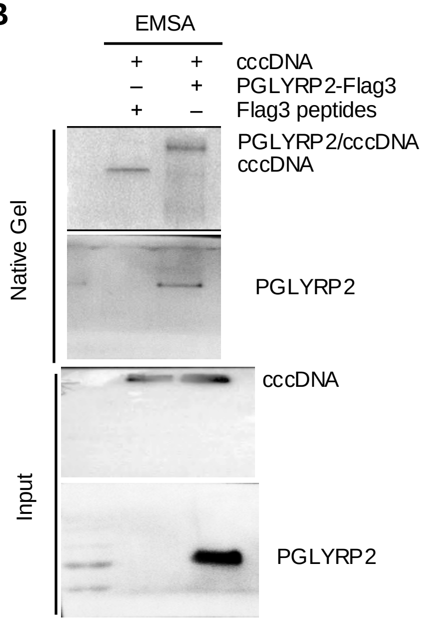

Full unedited blot for Figure 2L

D

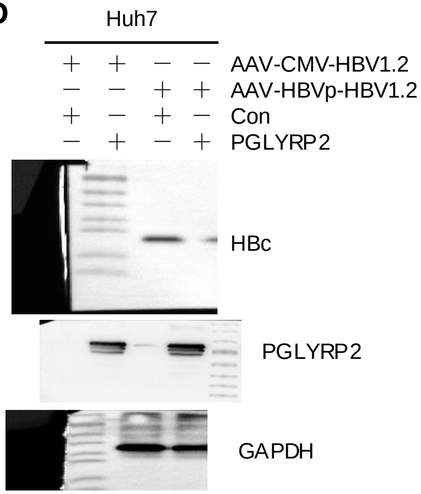

Full unedited blot for Figure S2D

C

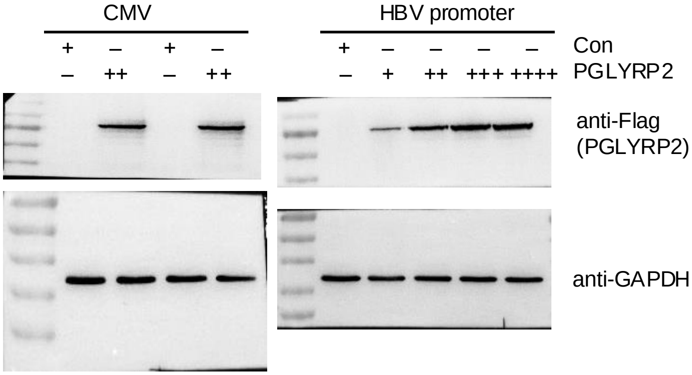

Full unedited blot for Figure S2A

E

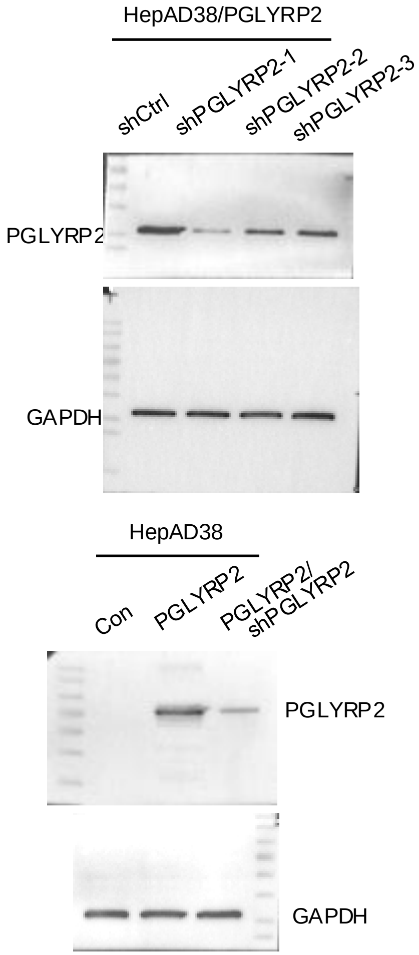

Full, unedited figures related to Figure 2 and Figure S2

Full unedited blot for Figure S2E

F

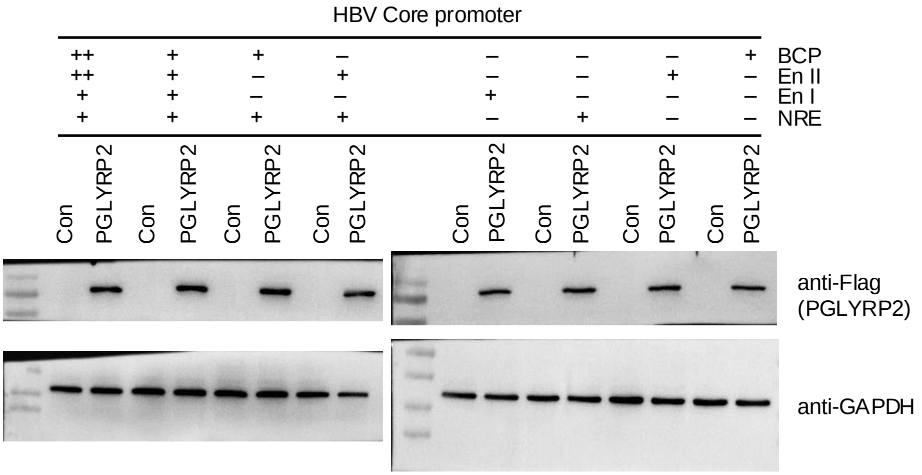

Full unedited blot for Figure S2B

G

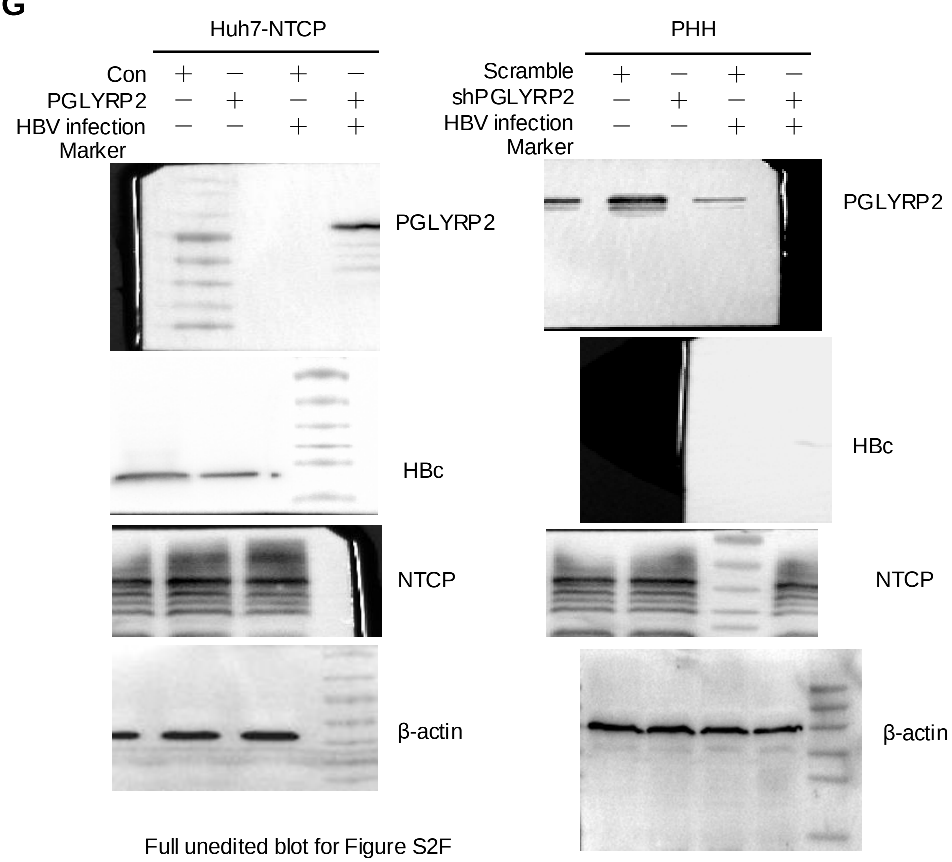

Full unedited blot for Figure S2F

Full, unedited figures related to Figure 2 and Figure S2, continued

A

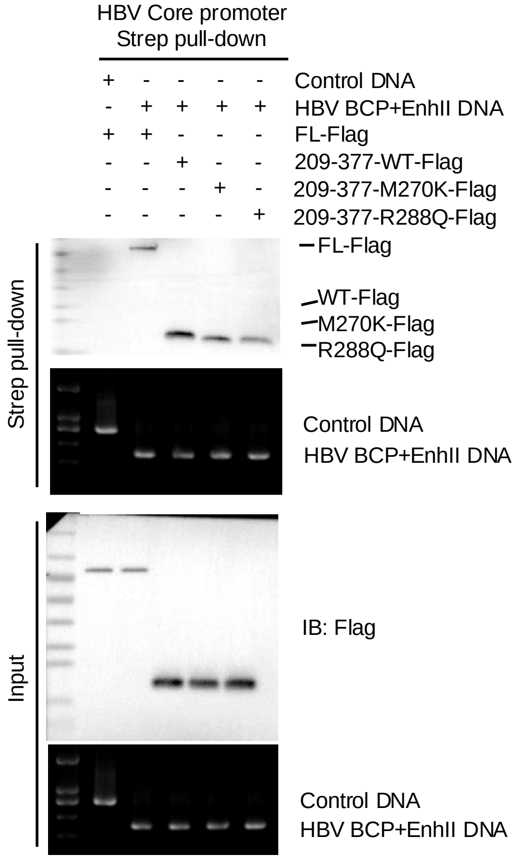

Full unedited blot/gel for Figure 3J

Full, unedited figures related to Figure 3 and Figure S3

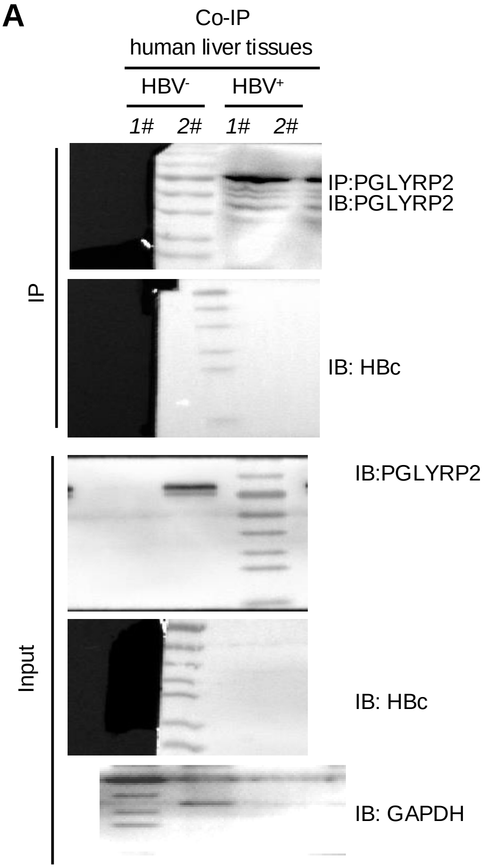

Full unedited blot for Figure 4B

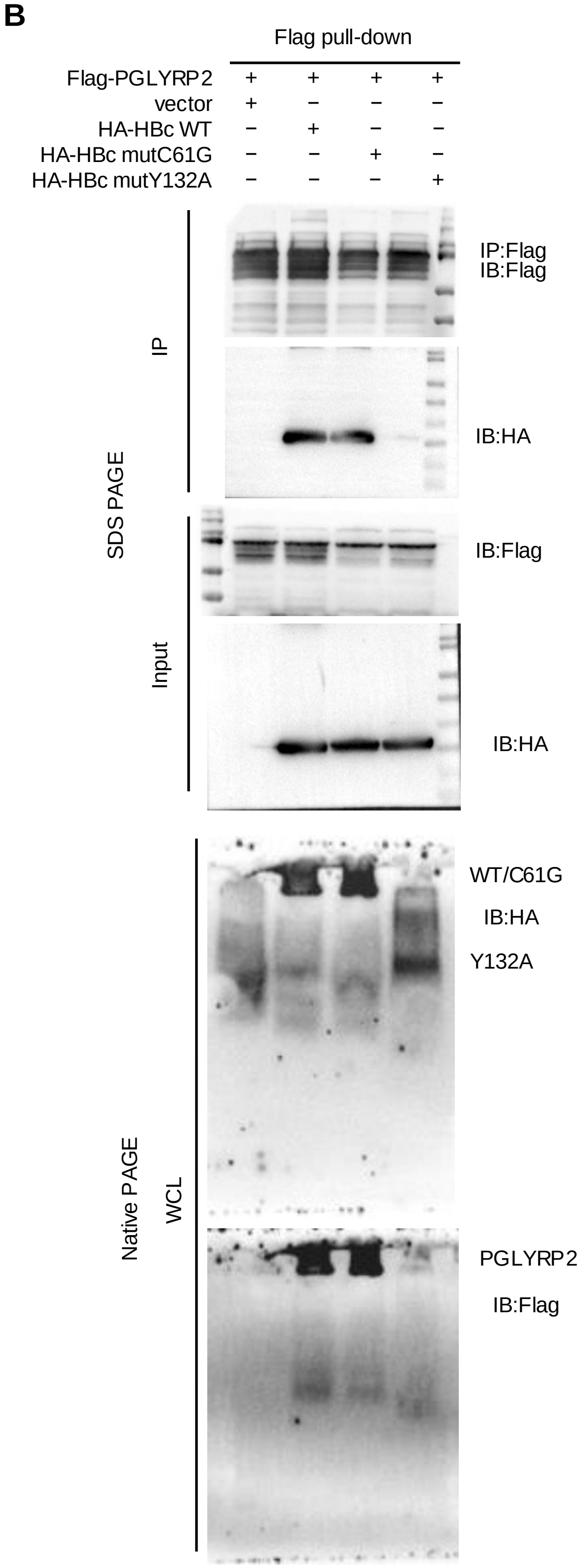

Full unedited blot for Figure 4D

Full, unedited figures related to Figure 4

A

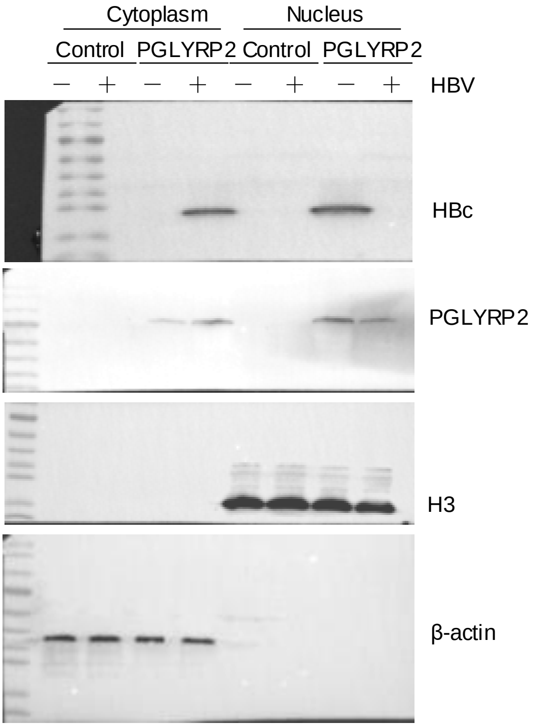

Full unedited blot for Figure S4A

B

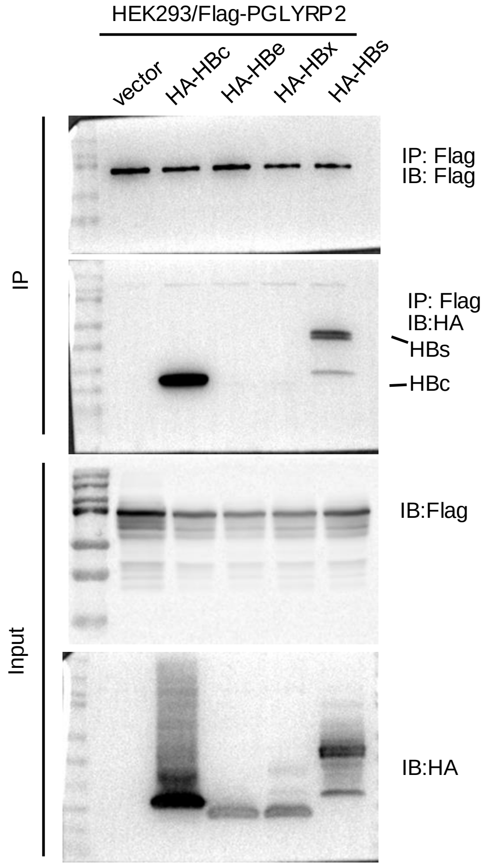

Full unedited blot for Figure S4B

C

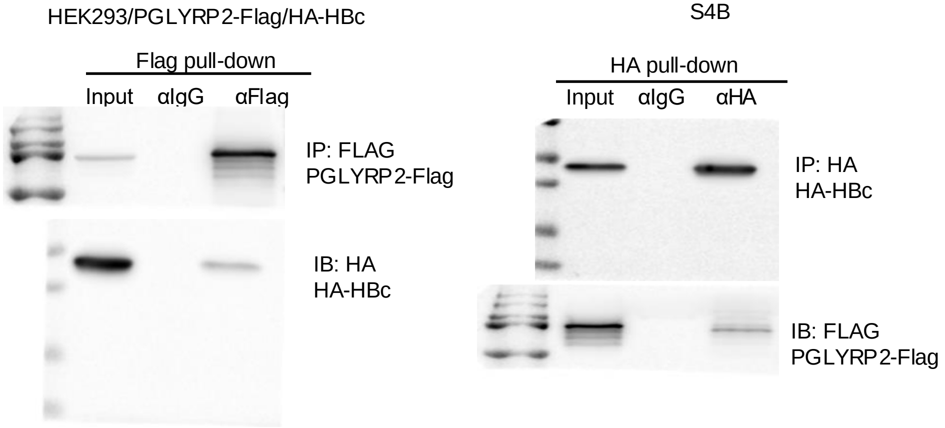

Full unedited blot for Figure S4C

Full, unedited figures related to Figure S4

D

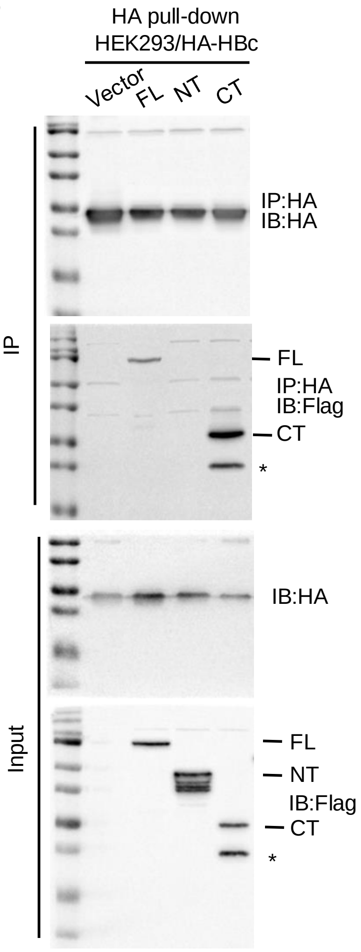

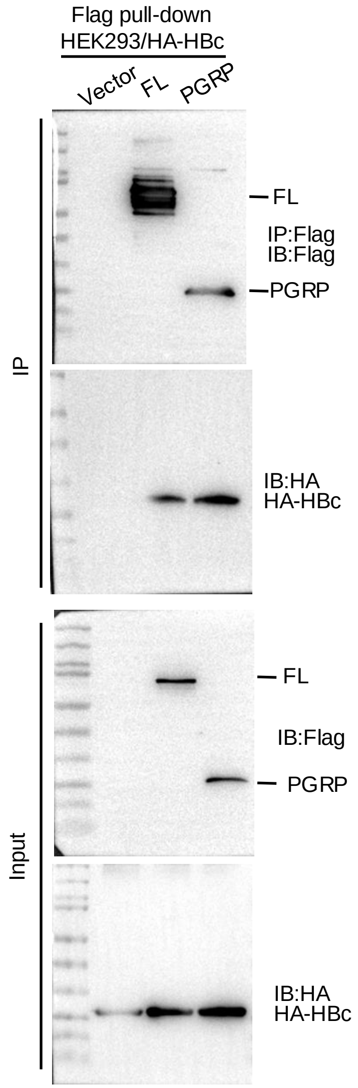

Full unedited blot for Figure S4D

E

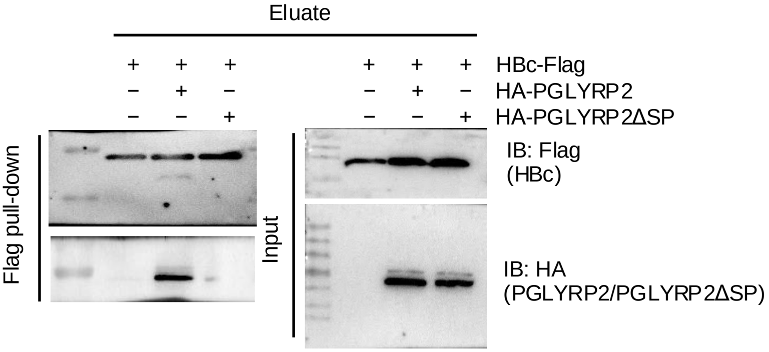

Full unedited blot for Figure S4E

Full, unedited figures related to Figure S4, continued

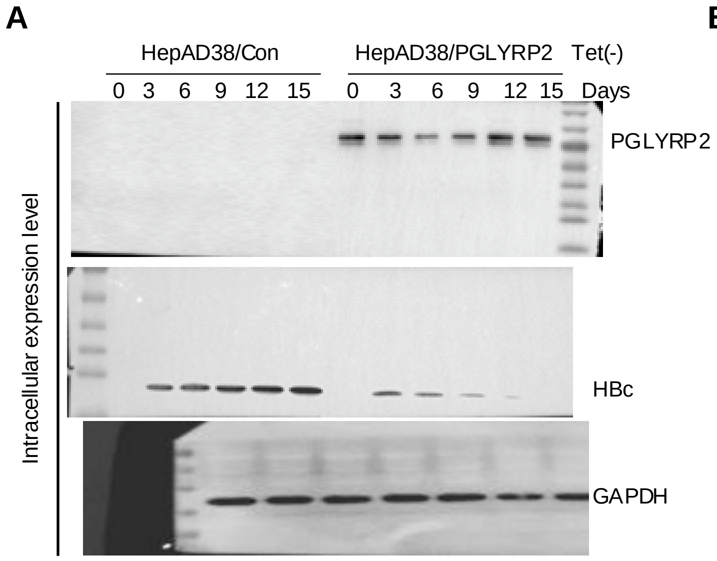

Full unedited blot for Figure 5C

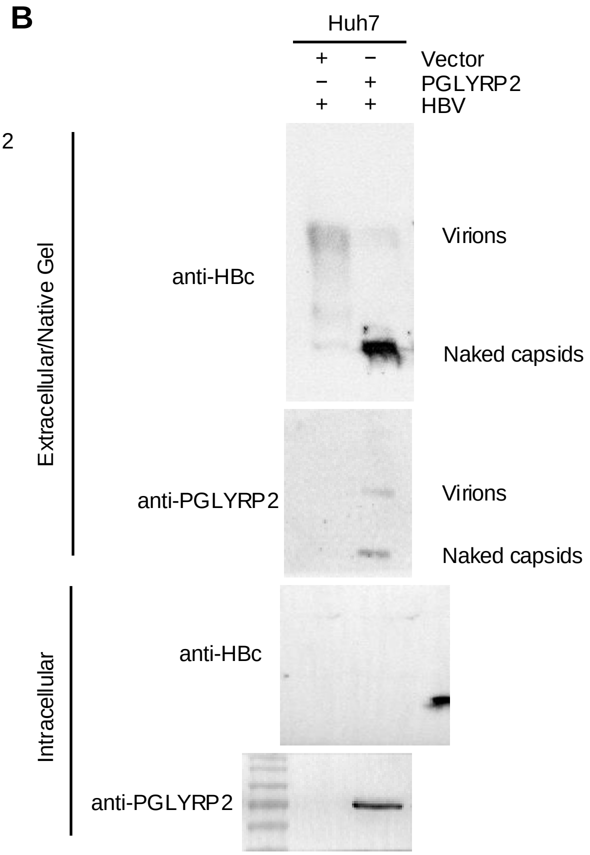

Full unedited blot for Figure 5E

Full, unedited figures related to Figure 5

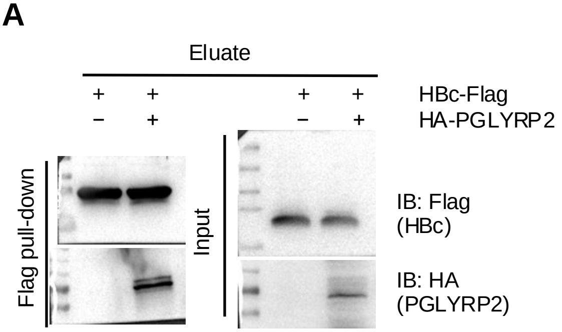

Full unedited blot for Figure 6A

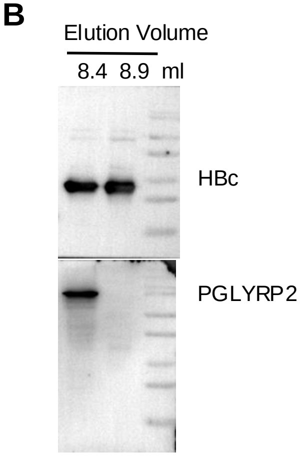

Full unedited blot for Figure 6B

Full, unedited figures related to Figure 6

A

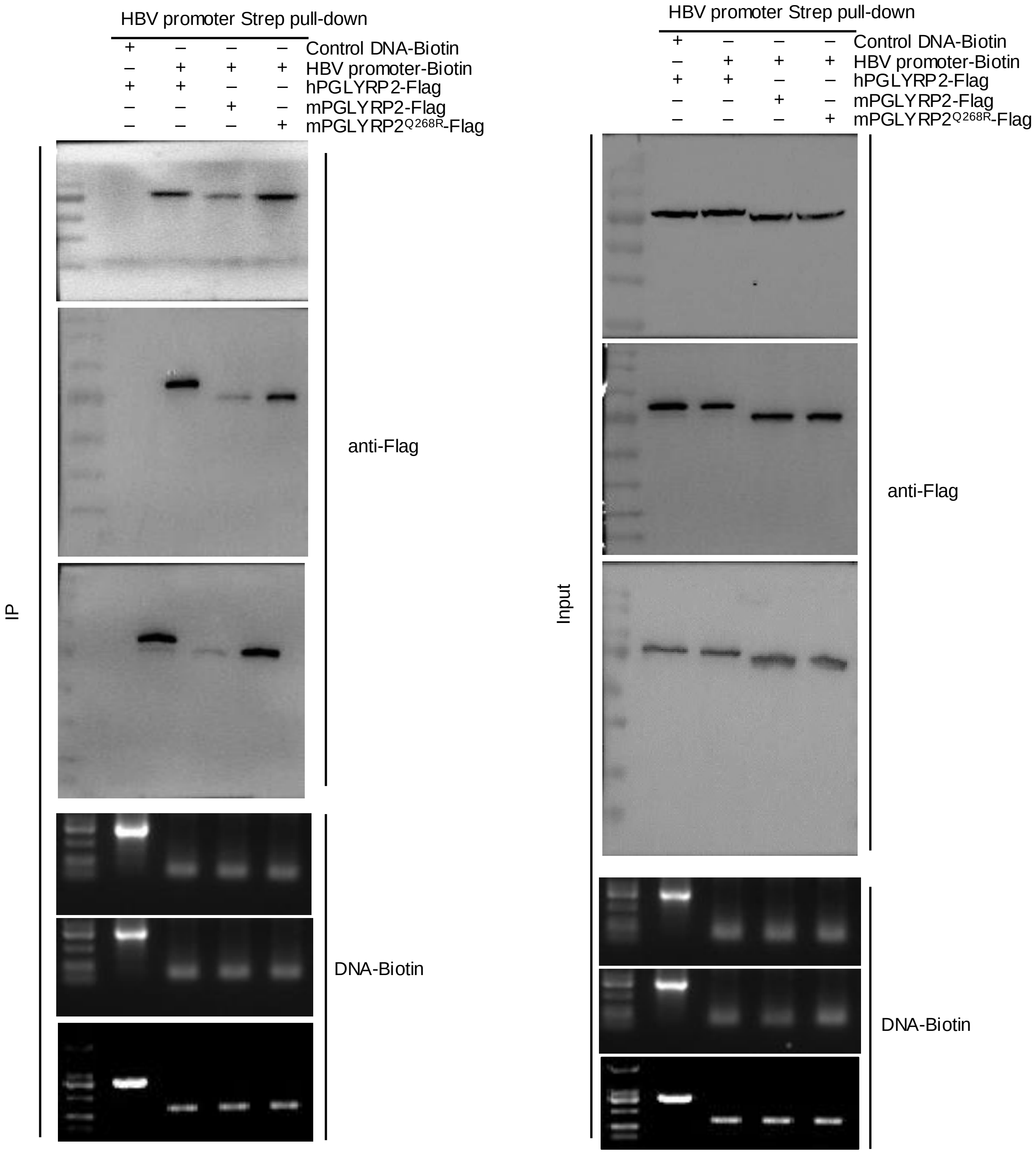

Full unedited blot for Figure 7C and Figure S7A

Full, unedited figures/gels related to Figure 7 and Figure S7
